# Antimetabolite pemetrexed primes a favorable tumor microenvironment for immune checkpoint blockade therapy

**DOI:** 10.1136/jitc-2020-001392

**Published:** 2020-11-26

**Authors:** Chia-Sing Lu, Ching-Wen Lin, Ya-Hsuan Chang, Hsuan-Yu Chen, Wei-Chia Chung, Wei-Yun Lai, Chao-Chi Ho, Tong-Hong Wang, Chi-Yuan Chen, Chen-Lin Yeh, Sean Wu, Shu-Ping Wang, Pan-Chyr Yang

**Affiliations:** 1 Department of Internal Medicine, National Taiwan University Hospital, National Taiwan University College of Medicine, Taipei, Taiwan; 2 Institute of Biomedical Sciences, Academia Sinica, Taipei, Taiwan; 3 Institute of Statistical Science, Academia Sinica, Taipei, Taiwan; 4 Tissue Bank, Chang Gung Memorial Hospital; Graduate Institute of Health Industry Technology and Research Center for Industry of Human Ecology, College of Human Ecology, Chang Gung University of Science and Technology, Taoyuan, Taiwan; 5 Institute of Biomedical Sciences and Genomics Research Center, Academia Sinica, Taipei, Taiwan

**Keywords:** immunotherapy, tumor microenvironment, drug therapy, combination, costimulatory and inhibitory t-cell receptors, tumor escape

## Abstract

**Background:**

The immune checkpoint blockade (ICB) targeting programmed cell death-1 (PD-1) and its ligand (PD-L1) has been proved beneficial for numerous types of cancers, including non-small-cell lung cancer (NSCLC). However, a significant number of patients with NSCLC still fail to respond to ICB due to unfavorable tumor microenvironment. To improve the efficacy, the immune-chemotherapy combination with pemetrexed, cis/carboplatin and pembrolizumab (anti-PD-1) has been recently approved as first-line treatment in advanced NSCLCs. While chemotherapeutic agents exert beneficial effects, the underlying antitumor mechanism(s) remains unclear.

**Methods:**

Pemetrexed, cisplatin and other chemotherapeutic agents were tested for the potential to induce PD-L1 expression in NSCLC cells by immunoblotting and flow cytometry. The ability to prime the tumor immune microenvironment was then determined by NSCLC/T cell coculture systems and syngeneic mouse models. Subpopulations of NSCLC cells responding differently to pemetrexed were selected and subjected to RNA-sequencing analysis. The key signaling pathways were identified and validated in vitro and in vivo.

**Results:**

Pemetrexed induced the transcriptional activation of *PD-L1* (encoded by *CD274*) by inactivating thymidylate synthase (TS) in NSCLC cells and, in turn, activating T-lymphocytes when combined with the anti-PD-1/PD-L1 therapy. Nuclear factor κB (NF-κB) signaling was activated by intracellular reactive oxygen species (ROSs) that were elevated by pemetrexed-mediated TS inactivation. The TS−ROS−NF-κB regulatory axis actively involves in pemetrexed-induced PD-L1 upregulation, whereas when pemetrexed fails to induce PD-L1 expression in NSCLC cells, NF-κB signaling is unregulated. In syngeneic mouse models, the combinatory treatment of pemetrexed with anti-PD-L1 antibody created a more favorable tumor microenvironment for the inhibition of tumor growth.

**Conclusions:**

Our findings reveal novel mechanisms showing that pemetrexed upregulates PD-L1 expression and primes a favorable microenvironment for ICB, which provides a mechanistic basis for the combinatory chemoimmunotherapy in NSCLC treatment.

## Background

Lung cancer is the leading cause of cancer-related death worldwide.^1^ Among non-small-cell lung cancer (NSCLC) represents a heterogeneous group of lung tumors based on histology and accounts for approximately 85% of lung cancers. Since the development of immune checkpoint blockade (ICB), immunotherapy has been state of the art in the treatment pipeline for patients with NSCLC in different stages of the disease.^2 3^ Several humanized monoclonal antibodies targeting programmed cell death-1 (PD-1) (eg, pembrolizumab and nivolumab) and programmed death-ligand 1 (PD-L1; eg, atezolizumab) have been approved by the US Food and Drug Administration (FDA) and been shown promising potencies as immunotherapeutic agents for NSCLC treatments.^4–6^ Although the PD‐1/PD‐L1 blockade therapy significantly improves the durable response rate and prolongs long-term survival for patients with NSCLC that lack targetable mutations, nearly 70% of patients in advanced stages of NSCLC are still unresponsive; especially those with low or no tumor PD-L1 expression.^7–10^ With impaired PD-L1 expression, tumors usually display a lack of T cell activation and/or infiltration in the surrounding tumor microenvironment.^11 12^ This kind of non-T-cell-inflamed tumor microenvironment has been characterized as ‘non-inflamed’ or ‘cold’ tumors. Thus, the clinical benefit from the PD-1/PD‐L1 blockade therapy in NSCLC treatment is limited, and the reactivation of PD-L1 expression in tumor cells could be a key factor for turning the cold tumor microenvironment into a ‘hot’ one.^13^ In this regard, identifying specific agents that show potentials to reactivate tumor PD-L1 expression may be critical in designing effective combinatory therapy regimens.^12^

The combination chemotherapy has long been the standard treatment for patients in advanced stages of NSCLC. Among the chemotherapeutic combinations, most commonly used are platinum-based agents, such as cisplatin or carboplatin, along with other chemotherapeutics. Emerging evidence indicates that modulation of the immune response through the PD-1/PD-L1 blockade can be enhanced by the potential immunogenic effects of cytotoxic chemotherapy, including increased tumor cell immunogenicity and repressed immunosuppressive circuitries.^14 15^ In accord with this view, several clinical trials are underway to evaluate the efficacy of the combination strategy with chemotherapeutics and immune checkpoint inhibitors.^16^ Pemetrexed (PEM), an antimetabolite that affects the folate pathway, is known to exert less side effects than cisplatin and carboplatin and is commonly used in the cisplatin/carboplatin-based combinatory chemotherapy for multiple solid tumors.^17^ In recent years, pemetrexed also shows huge potential in combination with immunotherapies.^18–20^ The combination of pemetrexed plus anti-PD-1 antibody (pembrolizumab), particularly in the presence of carboplatin-based chemotherapy, has been shown to improve the overall survival (OS) of patients with advanced stage NSCLC in KEYNOTE-021^19^ and KEYNOTE-189 trials.^20^ Recently, the IMpower132 trial also shows increased OS in the combination of pemetrexed plus anti-PD-L1 antibody (atezolizumab) in the presence of cisplatin or carboplatin.^21^ These improvements have led to the approval of the combination of pemetrexed, pembrolizumab (anti-PD-1), and platinum-based chemotherapeutics as the first-line treatment for patients with advanced non-squamous NSCLC. Nevertheless, it remains unclear how these chemotherapeutics enhance effects of cancer immunotherapy.

Here, we screen the effects of front-line NSCLC chemotherapeutic agents on PD-L1 expression in NSCLC cell lines and activation of tumor-infiltrating T-lymphocytes (TILs) in combination with ICB therapy. We find that sublethal doses of antimetabolites, pemetrexed and 5-fluorouracil (5-FU), can upregulate PD-L1 expression in NSCLC cells and modulate activities of TILs, priming a favorable tumor microenvironment for ICB therapy. By dissecting the mechanisms underlying the crosstalk between pemetrexed and ICB therapy, we further show that thymidylate synthase (TS) plays as a critical hub for connecting pemetrexed to the activation of NF-κB signaling pathway. Pemetrexed inhibits the activity of TS and in turn promotes the elevation of intracellular reactive oxygen species (ROSs), which enhances the phosphorylation of inhibitor of NF-κB (IκB) and subsequent activation of NF-κB signaling pathway. Thereby, this leads to the re-expression of PD-L1 in tumor cells, the augment and/or activation of TILs when combined with the PD-1/PD-L1 blockade, and the concomitant conversion of a cold tumor immune microenvironment to a hot state. These results provide mechanistic insights of pemetrexed in enhancing the anti-PD-1/PD-L1 therapy for NSCLC treatment.

## Methods

### Cell culture and inhibitors

The H1299 and A549 human lung cancer cell lines and the T47D human breast cancer cell line were obtained from the American Type Culture Collection (Manassas, Virginia) and cultured in Dulbecco’s Modified Eagle Medium containing 10% fetal bovine serum (FBS). The PC9 human lung cancer cell line was a gift from Dr C.-H. Yang (Graduate Institute of Oncology, Cancer Research Center, National Taiwan University). The CL141 and CL1-5 human lung cancer cell lines, derived from clinical patients, were established in our laboratory^22 23^ and maintained in Roswell Park Memorial Institute (RPMI) medium containing 10% FBS. Human peripheral blood mononuclear cells (PBMCs) were isolated from healthy blood donors. Isolation of PBMC in the buffy coats of fresh whole blood samples was performed by density gradient centrifugation on Ficoll-Paque Premium (GE Healthcare). Human PBMCs and Jurkat leukemia T cell line were cultured in RPMI medium containing 10% heat-inactivated FBS.

The IκB kinase (IKK) inhibitor BAY 11-7082 was acquired from MCE (MedChemExpress). Cell viability was analyzed by sulforhodamine B assay according to the manufacturer’s instructions. Further details about immunoblot analysis, real-time reverse transcriptase PCR, flow cytometry analysis, RNA interference and luciferase reporter assay are fully provided in the online supplemental materials.

10.1136/jitc-2020-001392.supp1 Supplementary data

10.1136/jitc-2020-001392.supp2 Supplementary data

### Xenograft tumor formation assay

CL1-5 cells (1×10^6^) were subcutaneously injected into the right flanks of 6-week-old nude mice, followed by intraperitoneal pemetrexed (50 mg/kg) every 4 days for two cycles. Tumors were collected 1 week after the final treatment. The isolated tumors were cut into small pieces, which were lysed with Radioimmunoprecipitation assay buffer (RIPA buffer, 1% Nonidet P-40, 0.5% sodium deoxycholate and 0.1% SDS) containing complete protease inhibitor cocktail (Roche). Protein samples were subjected to immunoblotting with antibodies against PD-L1 (#13684, Cell Signaling) and anti-β-actin (Sigma-Aldrich), the latter being the internal control.

### Interleukin-2 (IL-2) secretion by activated Jurkat T cells and PBMCs

CL1-5, CL141 and H1299 lung cancer cells were pretreated with chemotherapeutic drugs for 24 hours. These cells were subsequently cocultured with Jurkat T-cells or PBMCs at the cancer cell to T cell ratio of 5:1 (CL1-5 cells) or 10:1 (CL141 cells) for additional 48 hours in the presence of 1× T Cell Stimulation Cocktail (eBioscience). Coculture mediums were kept containing 1× T Cell Stimulation Cocktail and chemotherapeutic drugs. For blockade of the PD-1/PD-L1 pathway, 10 µg/mL of anti-PD-L1 antibody or control mouse IgG was added to the coculture medium. The culture supernatants were harvested at 48 hours after coculture and the concentrations of IL-2 were assessed by IL-2 Human ELISA Kit (Catalog # 88-7025-88, Thermo Scientific).

### Intracellular cytokine staining assays

For intracellular cytokine staining (ICS), monensin (3 µM) was added into coculture media for blocking cytokine secretion. Human Jurkat T-cells or PMBCs were harvested and washed with ice-cold PBS. Cells were then stained with specific antibodies for surface markers, CD45 (Biolegend, #368511) and CD69 (Biolegend, #310903), in cell staining buffer (Biolegend, #420201) for 30 min on ice in accordance with the manufacturer’s protocols. The intracellular staining of IL-2 (Biolegend, #500306) was performed with Cyto-Fast Fix/Perm buffer set (Biolegend, #426803). Afterwards, cells were washed twice with ice-cold PBS/FBS, resuspended, then analyzed by Fluorescence-activated cell sorting (FACS) analysis using a FACS Calibur system. FACS data analysis was performed using FlowJo software (Tree Star, Ashland, Oregon).

### T cell-mediated killing assay

CL141 lung cancer cells stably expressing NucLight red fluorescence protein (RFP) (#4476, Essen Bioscience) were seeded into 96-well plates and treated with anti-PD-L1 antibody (10 µg/mL) and/or pemetrexed (100 nM) for 24 hours. Cells were subsequently coincubated with activated Jurkat T-cells at the cancer cell to T cell ratio of 1:5. The fluorescence signals were detected by a SpectraMax iD3 fluorescence spectrometer (Molecular Devices).

### Syngeneic tumor model

For the CT26 tumor model, BALB/c mice aged 6–8 weeks were inoculated subcutaneously with murine CT26 (2×10^5^) colon cancer cells. For the LL2 tumor model, C57BL/6 mice aged 6–8 weeks were inoculated subcutaneously with murine LL2 (2×10^5^) lung cancer cells. Tumor volumes were measured at 4 or 5 days after inoculation using the formula: L × D^2^/2, where L and D are the long and short dimensions, respectively, of the tumor. Mice were injected intraperitoneally every 4 days with 3 mg/kg of antimouse PD-L1 antibody (10F.9G2, Bio X cell) or control rat IgG2b (LTF-2, Bio X cell). Mice were also injected intraperitoneally with 100 mg/kg of pemetrexed every 2 days for 2 weeks. Tumors were harvested on day 20 and fixed in formaldehyde for the immunohistochemistry (IHC) staining.

To assess the activation and/or infiltration of CD4^+^ and CD8^+^ TILs surrounding tumor tissues, tumors treated by monotherapy or combination therapy were harvest and analyzed by IHC staining. Details of analysis methods are fully provided in the online supplemental materials.

### RNA sequencing of the CL1-5 subclones

Using the limited dilution method, the parental CL1-5 lung cancer cells were seeded in the 96-well plate with an average of one cell per well. After 20 days of culture, subclones were expanded in RPMI-1640 medium supplemented with 10% FBS. Twenty single-cell colonies were selected as CL1-5 subclones and then treated with a sublethal dose of pemetrexed (50 nM). After 48 hours of treatment, the levels of PD-L1 protein were detected by immunoblotting. In treatment with pemetrexed (PEM), the CL1-5 subclones exhibiting elevated PD-L1 protein levels were defined as pemetrexed responders (PEM-R) while the subclones exhibiting no significant PD-L1 upregulation were defined as non-responders (PEM-NR).

For RNA sequencing and gene expression analysis, total RNA was extracted from pemetrexed-treated CL1-5 subclones. rRNA was depleted by the rRNA depletion method. Library preparation was followed by the manual protocol and paired-end reads with 150 bps were sequenced by Illumina HiSeq 4000 (Illumina). Sequencing quality and adaptor contamination were further evaluated by FASTQC (v0.11.7). Raw reads were cleaned by Trimmomatic (v0.33) for removal of adaptor sequences and lower quality bases (average quality score of 4-bases wide sliding window <20). Trimmed paired-end reads were aligned to human reference genome GRC37/hg19 using STAR2 (STAR-2.6.0c). Based on ensemble GRC37 gene annotation, gene expression was quantified by raw read counts using the htseq-count algorithm.

Before identifying differentially expressed genes between experiment groups, several preprocess steps were applied to avoid system errors. The read throughput difference was first adjusted by the trimmed mean of M-values algorithm of edgeR (R package). Next, count per million (CPM) was as gene expression unit. Genes with CPM<1 and not detected in at least two samples were excluded. Finally, logarithm 2 transformation and quantile normalization were applied before data analysis. For statistical analysis, the Log2 ratio of gene expression between the experiment and the corresponding control group was calculated. A two-sample t-test was used to identify differentially expressed genes. All tests were two-tailed and p values<0.05 were considered significant.

### ROS detection

Cells were trypsinized and resuspended in PBS containing 10 µM CM-H2DCFDA (#C6827; Invitrogen). After incubation at 37°C for 30 min in the dark, intracellular ROS levels were assessed by flow cytometry using a FACS Calibur system.

### Statistical analysis

Data are presented as the mean±SD; statistical signiﬁcance was assessed via GraphPad Prism Software V.7.0; groups were compared using the t-test, with p<0.05 considered signiﬁcant.

## Results

### Antimetabolites, pemetrexed and 5-FU, induce PD-L1 expression in NSCLC cells in vitro and in vivo

**Figure 1:**
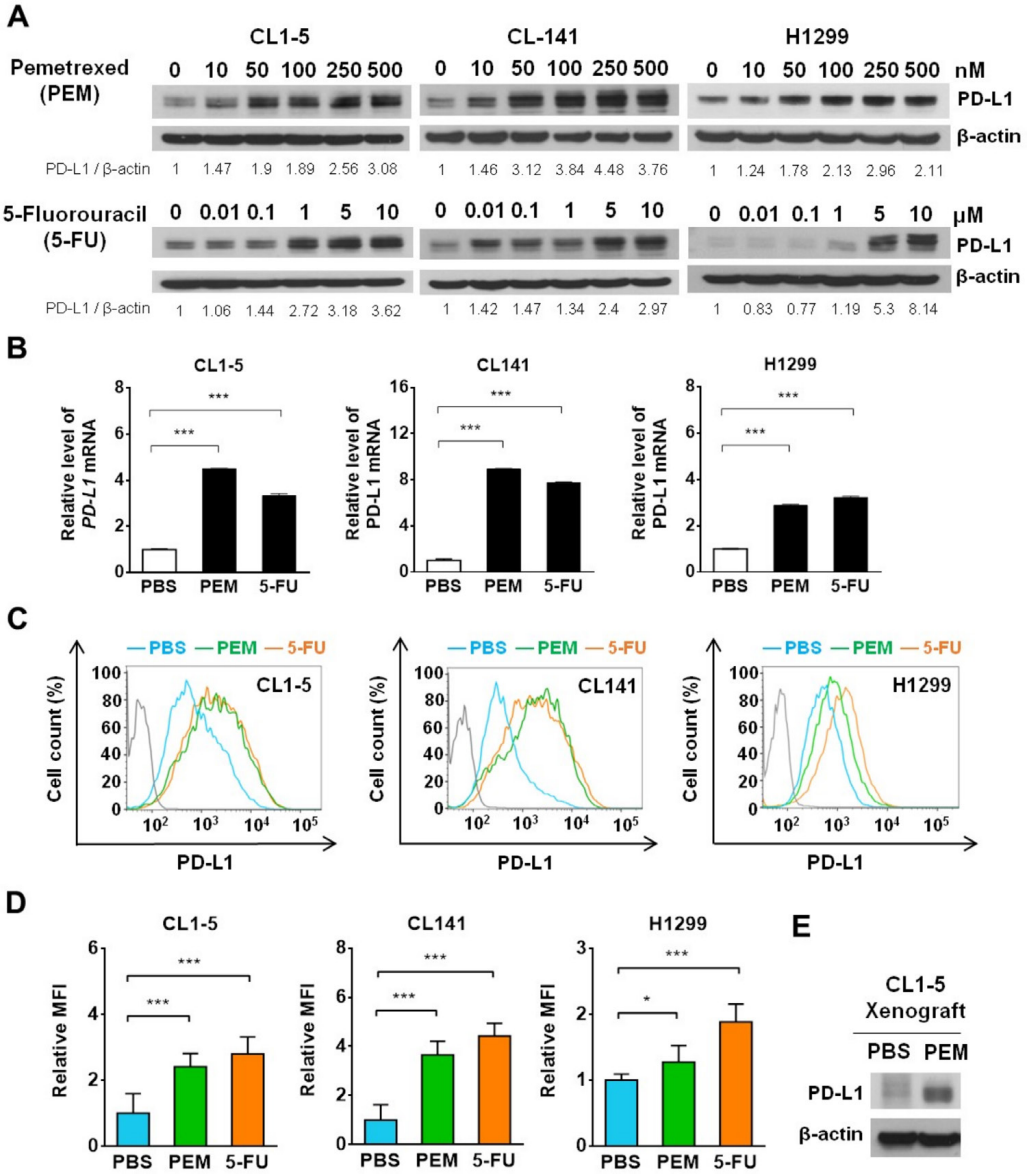
Pemetrexed (PEM) and 5-fluorouracil (5-FU) upregulate programmed cell death-ligand 1 (PD-L1) expression in non-small-cell lung cancer (NSCLC) cell lines and xenograft tumors. (A) Detection of PD-L1 in NSCLC cell lines CL1-5, CL141 and H1299 treated with sublethal concentrations of PEM, 5-FU, or the vehicle control (PBS) for 72 hours by immunoblotting. The expression of β-actin serves as a loading control. The ratios between the intensity of the bands corresponding to programmed death-ligand 1 (PD-L1) and those corresponding to β-actin were calculated. (B–D) CL1-5, CL141 and H1299 cells were treated with PBS, 100 nM PEM or 5 µM 5-FU for 72 hours. Relative *PD-L1* mRNA expression levels, determined by qRT-PCR, are shown in (B). Representative histograms for PD-L1 expression levels on the cell surface of NSCLC cells obtained by flow cytometry are shown in (C). Relative mean fluorescence intensity for PD-L1 expression levels is shown in (D). (E) CL1-5 cells were subcutaneously inoculated into nude mice and intravenously injected with PEM. The tumors were harvested and PD-L1 expression levels were assessed by immunoblotting. All the data are shown as means and s.e.m. for three independent experiments (n=3). *P<0.005 and ***p<0.001 by Student’s t test.

Antitumor therapy becomes more successful when it can induce an immunogenic form of tumor cell death (ICD) that elicits a strong immune response.^24 25^ Recently, monotherapy of the frontline NSCLC chemotherapeutics, such as pemetrexed, carboplatin and paclitaxel (PTX), have been shown to induce ICD in syngeneic mouse models with various degrees while, interestingly, only the folate antimetabolite pemetrexed show the greater potential to synergize with PD-1/PD-L1 blockade.^26^ Although pemetrexed exerts the strongest immunogenic effects on tumor and immune cells, we noticed that pemetrexed also induced substantial levels of PD-L1 upregulation.^26^ Knowing that higher PD-L1 expression on tumor cells associated with better responses to ICB therapy,^27^ we attempted to assess the potential PD-L1 priming ability of pemetrexed and other frontline NSCLC chemotherapeutics in NSCLC cell lines. The half-maximal inhibitory concentration (IC_50_) of pemetrexed (PEM), 5-FU, cisplatin, and PTX were initially determined in the commonly used NSCLC cell lines CL1-5, CL141 and H1299^22 23^ (online supplemental table S1). To avoid triggering massive tumor cell death, these NSCLC cell lines were treated with the corresponding sublethal doses of chemotherapeutics for 72 hours and examined for their PD-L1 levels. Interestingly, we found that protein levels of PD-L1 were elevated by pemetrexed or 5-FU in a dose-dependent manner (figure 1A), while treatment with cisplatin or PTX did not increase PD-L1 expression (online supplemental figure S1A). We further confirmed that upregulation of PD-L1 (encoded by *CD274*) by either pemetrexed or 5-FU was initiated at the transcriptional level (figure 1B). To assess whether the pemetrexed and 5-FU-induced *PD-L1* gene upregulation produces functionally and clinically targetable PD-L1 proteins, we detected the membrane-bound PD-L1 protein by anti-PD-L1 flow cytometry antibodies. NSCLC cells treated with pemetrexed or 5-FU displayed a significant increase in PD-L1 levels on the cell surface (figure 1C, D), whereas cisplatin and PTX did not give such PD-L1 priming effects (online supplemental figure S1C, D). To further confirm this indication, we assessed the pemetrexed-mediated PD-L1 priming effect in human xenograft tumors. Human CL1-5 lung cancer cells were implanted into nude mice to form subcutaneous tumors followed by the injection of pemetrexed. We found that mice treated with pemetrexed displayed a substantial increase in PD-L1 levels in the CL1-5 xenograft tumor (figure 1E), suggesting that pemetrexed may stimulate certain signaling(s) in NSCLC tumors that drive the upregulation of PD-L1. Collectively, these results suggest that the sublethal dose of antimetabolic chemotherapeutics, such as pemetrexed and 5-FU, is capable of inducing PD-L1 expression in NSCLC cells whether in vitro or in vivo.

### Pemetrexed and PD-1/PD-L1 blockade induce T-cell activation in cocultured NSCLC and T cells

**Figure 2:**
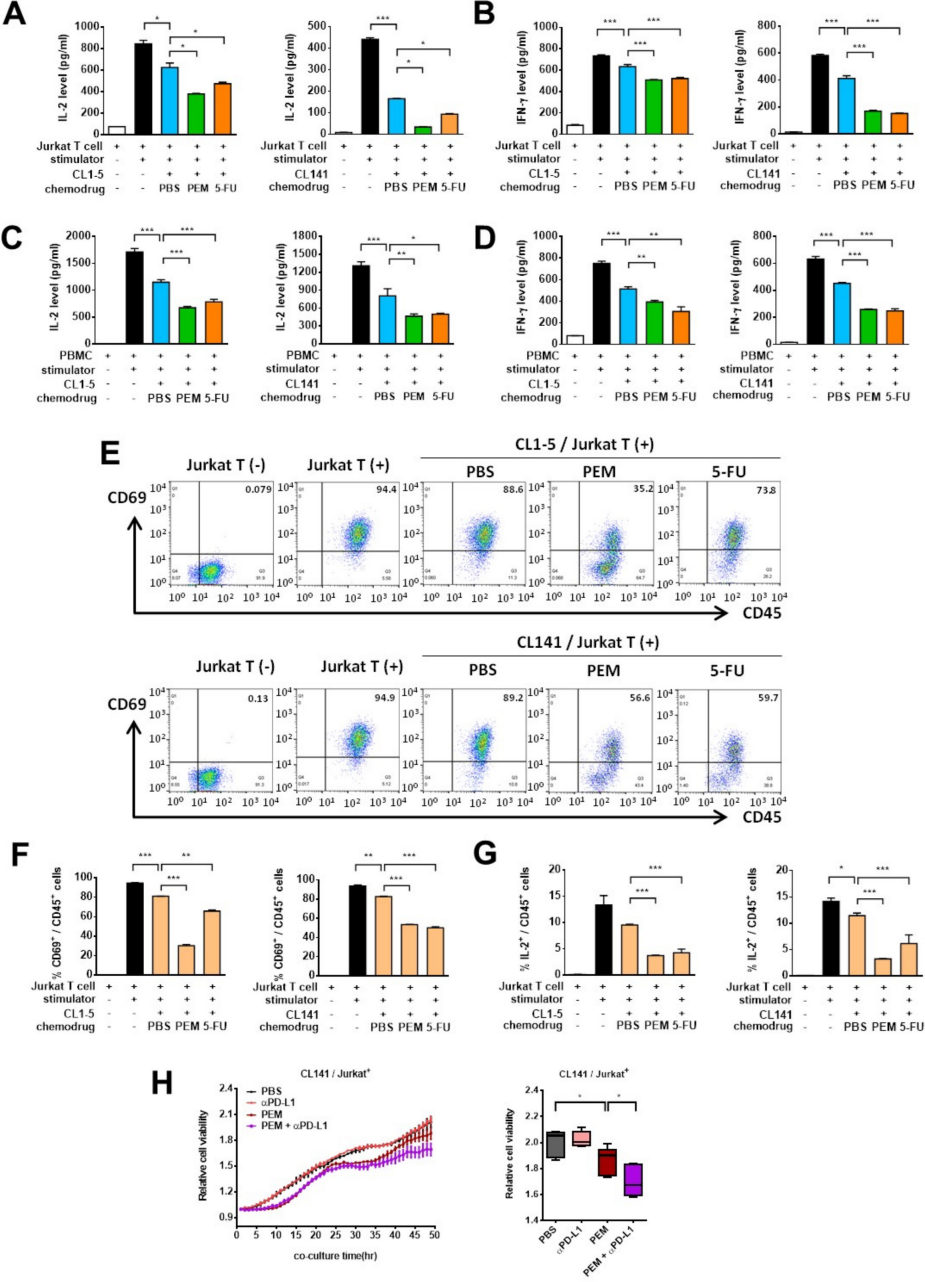
Pemetrexed (PEM) and 5-fluorouracil (5-FU) suppress the production of interleukin-2 (IL-2) and interferon (IFN)-γ by activated T cells in the non-small-cell lung cancer (NSCLC) and T cell coculture system. (A–D) CL1-5 or CL141 cells were preincubated with 100 nM PEM, 5 µM 5-FU or the vehicle control (PBS) for 48 hours, and subsequently cocultured with activated Jurkat T-cells (A, B) or PBMCs (C, D) at different cancer to T cell ratios in the presence of 1× T cell stimulation cocktail for additional 48 hours. The levels of IL-2 and IFN-γ were measured by ELISA. Data are shown as means and SD for three independent experiments (n=3). (E–G) CL1-5 or CL141 cells were preincubated with 100 nM PEM, 5 µM 5-FU or the vehicle control (PBS) for 48 hours, and subsequently cocultured with activated Jurkat T-cells in the presence of 1× T cell stimulation cocktail for additional 48 hours. The levels of CD69 and intracecllular IL-2 produced by Jurkat T-cells were measured by flow cytometry. (E) Representative dot plots for the indicated cell percentages determined by flow cytometry. (F, G) Quantitative plots for the CD69 and intracellular IL-2 staining in CD45^+^ T-cells. (H) T-cell-meditated killing of PD-L1-expressing CL141 NSCLC cells. CL141 cells stably expressing nuclear RFP protein were pretreated with or without 50 nM PEM for 48 hours and cocultured with activated Jurkat T-cells with or without 10 µg/mL of anti-PD-L1 antibody for additional 48 hours. Relative cell viability was measured by RFP signaling after 48 hours of coincubation and the results were normalized at the zero-time point. Data are shown as means and s.e.m. for three independent experiments (n=3). *P<0.005, **p<0.01 and ***p<0.001 by Student’s t-test.

The chemoimmunotherapy combination of pemetrexed, cis/carboplatin and immune checkpoint inhibitors (anti-PD-1/anti-PD-L1) has recently been approved as first-line treatment in advanced NSCLCs.^19–21^ However, which chemotherapeutics in the chemoimmunotherapy combination exert beneficial effects and the underlying antitumor mechanism(s) still remain obscure. T-cell activation is a key indicator of ICB therapy, which can be assessed by monitoring well-known surface markers (eg, CD69) and cytokine molecules (eg, IL-2, IFN-γ) enriched in activated T cells in human cytotoxic T lymphocyte (CTL) or Jurkat leukemia T cell coculture systems.^28^ To evaluate whether treatment of NSCLC cells with frontline NSCLC chemotherapeutics can modulate T-cell activation, we measured the levels of secreted IL-2 and IFN-γ in human Jurkat T-cells or PBMCs, which include CD3^+^ T-lymphocytes, incubated either alone or in coculture with NSCLC cells. The ELISA showed that activated Jurkat T-cells or PBMCs secreted high amounts of IL-2 and IFN-γ into the coculture medium, whereas the ability of the T cells to produce these antitumor cytokines was substantially suppressed when cocultured with CL1-5 or CL141 cells (figure 2A–D). This is consistent with previous findings that tumor cells may hijack the immune checkpoints to inhibit or exhaust T-cell activities through the PD-1/PD-L1 pathway.^29^ Indeed, CL1-5 and CL141 cells pretreated with pemetrexed or 5-FU displayed greater abilities to inhibit Jurkat T-cell or CTL-mediated cytokine secretion (figure 2A–D), supporting the idea that these antimetabolic chemotherapeutics are capable of inducing PD-L1 expression in tumor cells and the following T-cell suppression. In contrast, there were no detectable changes in IL-2 secretion in T cells when NSCLC cells were pretreated with chemotherapeutics (ie, cisplatin and PTX) showing no effects on PD-L1 upregulation (online supplemental figure S2A). The ICS assays further confirmed that NSCLC cells pretreated with pemetrexed or 5-FU greatly suppressed the levels of CD69 and intracellular IL-2 in human Jurkat T-cells (figure 2E–G and online supplemental figure S2C, D). To further test whether pemetrexed in combination with ICB therapy could exert antitumor activities through T-cell activation, we evaluated effects of the combined treatment of pemetrexed and anti-PD-1 antibody on T-cell activation in the NSCLC/T-cell coculture system. We found that addition of anti-PD-1 antibody or anti-PD-L1 antibody in pemetrexed-treated coculture system greatly reinvigorates the exhausted T cells by blocking the PD-1/PD-L1 interactions (online supplemental figure S2B). The effects of the combined treatment of pemetrexed and anti-PD-L1 antibody on the tumor-killing activity of T cells were also tested. CL141 cells stably expressing a nuclear RFP, NucLight RFP, were cocultured with activated Jurkat T-cells in the presence of pemetrexed and/or anti-PD-L1 antibody. By measuring the RFP signals, which represent cell viability, we found that sublethal dose of pemetrexed only caused partial suppression of tumor cell viability (figure 2H). However, the combined treatment of pemetrexed and anti-PD-L1 antibody resulted in the synergistic inhibition of tumor cell growth (figure 2H). These findings indicate that while pemetrexed could only exert minor cytotoxic effects on tumor cells, pemetrexed-induced PD-L1 upregulation plus the subsequent PD-1/PD-L1 blockade could sensitize tumor cells to the T cell-mediated tumor cell killing. Altogether, our data suggest that pemetrexed or 5-FU-induced PD-L1 upregulation in NSCLC cells can modulate T-cell activation when combined with PD-1/PD-L1 blockade, while addition of cisplatin or PTX did not have such immunomodulatory effects.

### Pemetrexed and PD-1/PD-L1 blockade synergistically inhibit tumor growth, increase PD-L1 expression, recruit TILs, and restore exhausted T-cell activities

**Figure 3:**
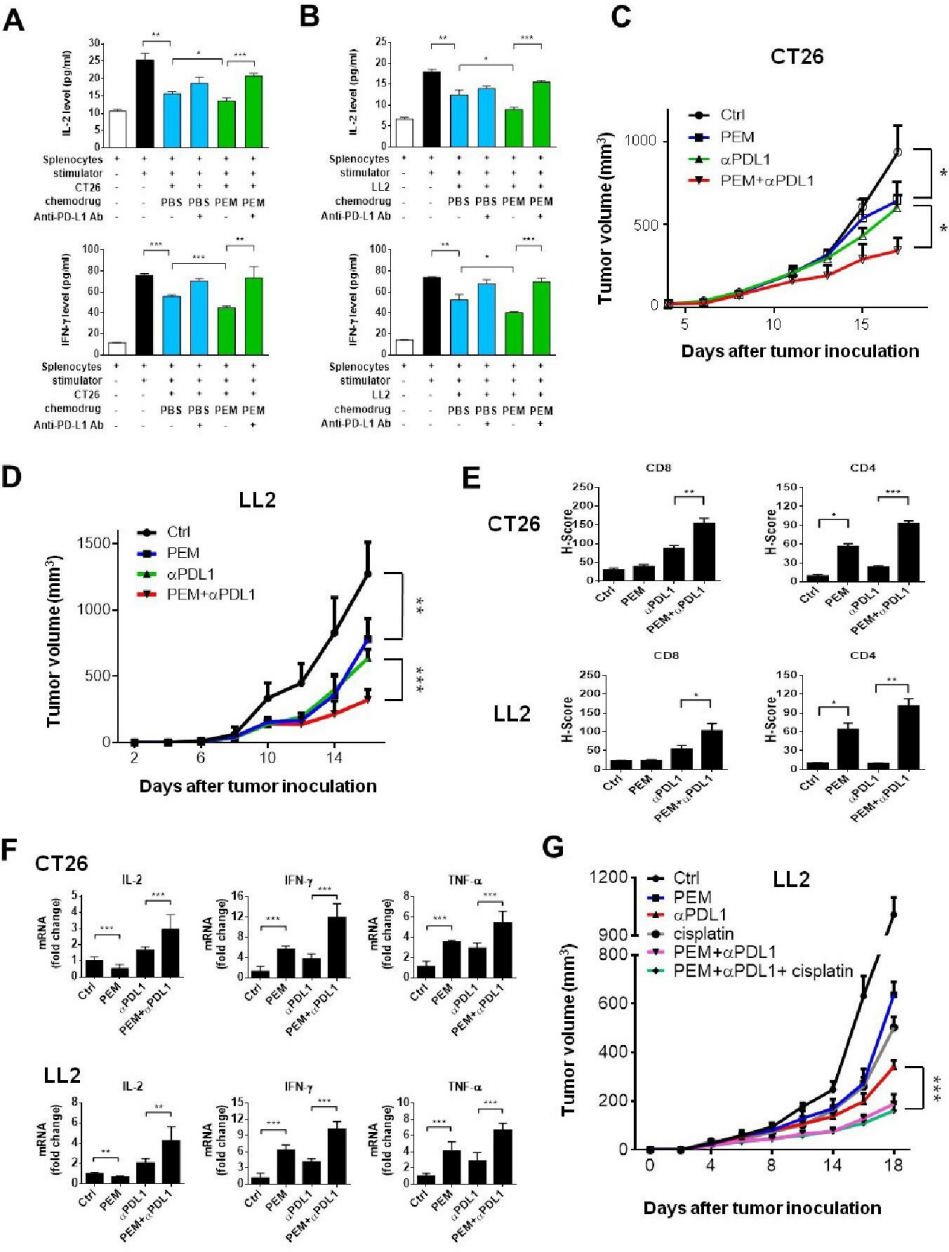
Pemetrexed (PEM) promotes the efficacy of anti-programmed death-ligand 1 (PD-L1) antibody in tumor cell killing in mouse syngeneic tumor models. (A, B) Spleen was removed from wild mouse immediately after killing; erythrocytes were then lysed, and the spleen-derived lymphocytes from each mouse were then resuspended in RPMI 1640 with 10% heat-inactivated fetal bovine serum. CT26 and LL2 cells were preincubated with 100 nM PEM or the vehicle control (PBS) for 48 hours, and subsequently cocultured with activated spleen-derived lymphocytes in the presence of 1× T cell stimulation cocktail with or without anti-programmed cell death-ligand 1 (PD-L1) antibody (10 µg/mL) for additional 48 hours. The levels of interleukin-2 (IL-2) and interferon (IFN)-γ were measured by ELISA. Data are shown as means and SD for three independent experiments (n=3). (C–F) The combinational treatment of PEM and anti-PD-L1 antibody inhibits tumor growth in the CT26 and LL2 mouse tumor models. Murine CT26 cells were implanted into Balb/c mice and LL2 cells were implanted into C57BL/6 mice based on their origins. Tumor volumes were measured at the indicated time points and shown in C (CT26) and D (LL2). The positive staining signals from the immunohistochemistry of CD4 and CD8 in serial sections of tumor specimens were quantified by the H-score system and shown in (E). (F) Relative mRNA expression levels of antitumor cytokines IL-2, tumor necrosis factor (TNF)-α and IFN-γ in the tumor area. Total RNA was extracted from tumors in each group and relative mRNA expression levels of IL-2, TNF-α and IFN-γ were determined by qRT-PCR. Data are shown as means and SD for three independent experiments (n=3). (G) Effects of different combinations of PEM (100 mg/kg), cisplatin (4 mg/kg), and anti-PD-L1 antibody (3 mg/kg) on tumor growth in the LL2 mouse tumor model.

The observed immunomodulatory effects of pemetrexed in combination with PD-1/PD-L1 blockade in the in vitro system prompted us to evaluate this chemoimmunotherapy combination in immunocompetent syngeneic mouse tumor models. We chose the commonly used murine Lewis lung carcinoma (LL2) and colorectal cancer cell (CT26) lines and confirmed that both cell lines respond to the treatment with pemetrexed or 5-FU by membrane-bound PD-L1 protein upregulation (online supplemental figure S3A). To test the effects of pemetrexed on increasing the efficacy of ICB therapy in the mouse tumor models, we cocultured mouse LL2 or CT26 cancer cell lines with primary splenocytes derived from the mouse spleen and measured the levels of secreted IL-2 and IFN-γ in the coculture system. Similar to the effects in human NSCLC/T cell coculture systems, we found that pemetrexed treatment significantly suppresses the splenocyte-mediated cytokine secretion (figure 3A, B). Notably, the addition of anti-PD-L1 antibody in pemetrexed-treated coculture system greatly induced splenocyte activation (figure 3A, B), confirming that the effects of the pemetrexed/anti-PD-L1 antibody combinational therapy can also be observed in the mouse tumor models. Moreover, the number of CD3^+^ T-lymphocytes, which contains CD4^+^ helper and CD8^+^ cytotoxic T-cells, in the splenocyte population was greatly increased in the combinational treatment of pemetrexed and anti-PD-L1 antibody (online supplemental figure S3B). The effects of the pemetrexed/anti-PD-L1 antibody combinational therapy on tumor growth were further evaluated by monitoring the tumor sizes and the surrounding tumor microenvironment in mouse syngeneic tumor models. Although treatment with pemetrexed or anti-PD-L1 antibody showed significant tumor growth hindrance in both mouse models (ie, LL2 and CT26), the combined treatment exerted far more superior tumor suppressive effects (figure 3C, D). The tumors were harvested and assessed for PD-L1 expression and the recruitment and/or activation of CD4^+^ and CD8^+^ TILs. The IHC staining in tumor specimens showed substantial PD-L1 signals in tumors receiving pemetrexed (online supplemental figure 3C, D). IHC staining of CD4 in tumor specimens showed that pemetrexed treatment induced a significant increase in the number of CD4^+^ TILs in tumor areas (figure 3E), suggesting that the tumor cytotoxic effects of pemetrexed may trigger the activation and/or recruitment of CD4^+^ T helper cells to the tumors. Through the IHC staining, we also found that the anti-PD-L1 antibody triggered the activation and/or infiltration of CD8^+^ T cells into tumors (figure 3E), consistent with the concept of ICB therapy. Interestingly, the combined treatment of pemetrexed and anti-PD-L1 antibody synergistically amplified the numbers of both CD4^+^ and CD8^+^ TILs even more in the tumor surroundings (figure 3E), indicating that the immunosuppressive tumor microenvironment has been altered through this combinational treatment. To further confirm this idea, we detected the expression levels of various antitumor cytokines, tumor necrosis factor (TNF)-α, IFN-γ and IL-2, that have been shown to lose their expressions in exhausted TILs^30 31^ and found that this combinational treatment induced much higher levels of IL-2, TNF-α and IFN-γ in tumor areas than individual drug treatments (figure 3F).

To test the therapeutic effect platinum-based chemotherapy has when combined with pemetrexed and ICB therapy in NSCLC treatment, we investigated whether the addition of cisplatin into the combinational treatment of pemetrexed and anti-PD-L1 antibody would exhibit an enhanced effect on the suppression of lung tumor growth in vivo. Although monotherapy with pemetrexed, cisplatin, or anti-PD-L1 antibody all showed significant tumor growth inhibition in the LL2 mouse lung tumor model, the combined treatment of pemetrexed and anti-PD-L1 antibody exerted far more superior tumor suppressive effects (figure 3G). Interestingly, there seems to be no advantage of cisplatin in the combination therapy of pemetrexed and anti-PD-L1 antibody for LL2 lung cancer treatment (figure 3G). Thus, our results indicate that the combination of antimetabolic chemotherapeutics (eg, pemetrexed) with PD-1/PD-L1 blockade (eg, anti-PD-L1 antibody) could further enhance the antitumor immune responses and restore exhausted T-cell activities, thereby priming a favorable tumor microenvironment for cancer immunotherapy.

### RNA-sequencing for NSCLC subpopulations identifies NF-κB signaling involved in pemetrexed-induced PD-L1 upregulation

**Figure 4:**
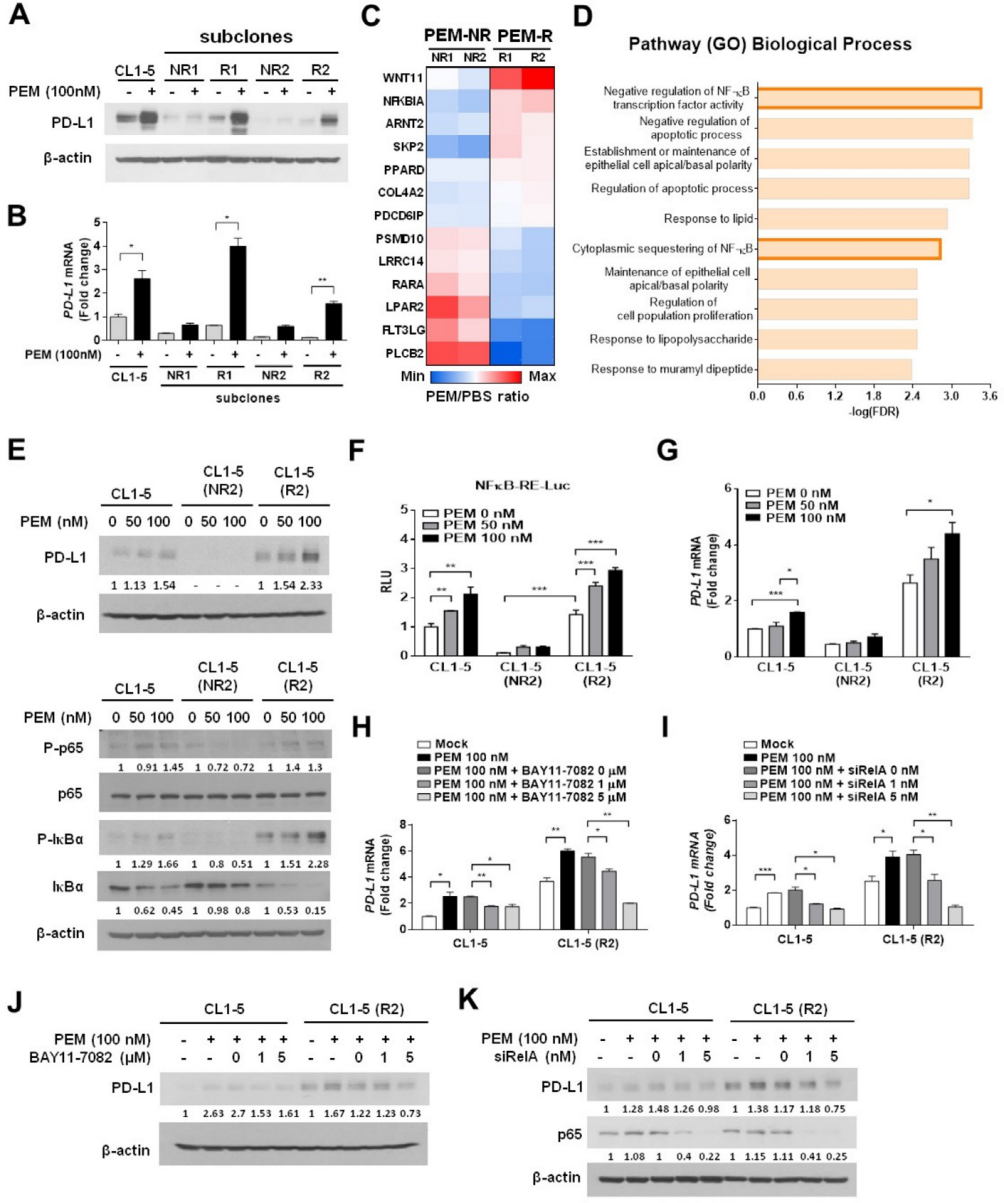
NF-κB signaling acts downstream of pemetrexed (PEM) to induce the transcriptional activation of programmed death-ligand 1 (*PD-L1)* in non-small-cell lung cancer (NSCLC) cells. (A, B) PD-L1 expression levels in two groups of CL1-5 cell subclones, PEM responder (PEM-R) and PEM non-responder (PEM-NR), in response to PEM incubation. CL1-5 subclonal cells were treated with 100 nM PEM or the vehicle control (PBS) for 72 hours. The protein levels of PD-L1 were detected by immunoblotting and shown in (A). The levels of β-actin serve as a loading control. Relative *PD-L1* mRNA expression levels, determined by qRT-PCR, are shown in B. Data are shown as means and s.e.m. for three independent experiments (n=3). (C) Heat map of representative cancer-related genes (p<0.05) from RNA-seq results between PEM-R and PEM-NR groups were identified using Kyoto Encyclopedia of Genes and Genomes (KEGG) analysis. Red and blue colors indicate increased and decreased levels, respectively. (D) The top 10 most significant terms or pathways were revealed by Search Tool for the Retrieval of Interacting Genes/Proteins (STRING) analysis. The bars represent the adjusted p value (FDR). (E) CL1-5, CL1-5/NR2 and CL1-5/R2 cells were treated with 50 or 100 nM PEM or the vehicle control (PBS) for 72 hours. The expression of the indicated total or phosphorylated proteins was evaluated by immunoblotting. The levels of β-actin serve as a loading control. (F) Transcriptional activation of a NF-κB reporter gene in PEM-treated parental and subclonal CL1-5 cells. NF-κB—Luc, a NF-κB responsive promoter–reporter construct, was transfected into each CL1-5 cell clone as indicated. The luminescence signal from the NF-κB—Luc reporter (firefly luciferase) was normalized to that of cotransfected pRL-TK vector (Renilla luciferase) to control for transfection efficiency and was expressed as relative light units. Data are shown as means and SD for three independent experiments (n=3). (G) Relative mRNA expression levels of *PD-L1,* determined by qRT-PCR, in each CL1-5 cell clone in response to PEM. (H) Inhibition of NF-κB signaling impairs the PEM-induced PD-L1 upregulation. Each CL1-5 cell clone was cotreated with PEM (100 nM) and different doses of the IKK inhibitor (BAY117082) for 3 days. Total RNA was isolated and relative *PD-L1* mRNA levels were determined by qRT-PCR. The same experiment was performed and the protein levels of PD-L1 were evaluated with immunoblotting (J). Numbers below the respective panels of the immunoblots indicate the densitometric values normalized with the relative β-actin value. (I) Knockdown of NF-κB p65 reverses the effects of PEM on the expression of PD-L1. CL1-5 or CL1-5/PEM-R2 cells were transfected with control-siRNA (siCtrl) or *RelA*-siRNA (siRelA) oligonucleotides for 24 hours and followed by treatment with 100 nM PEM or the vehicle control (PBS) for additional 72 hours. Cells were lysed and analyzed by qRT-PCR (I) or immunoblotting (K). Data are shown as means and SD for three independent experiments (n=3). *P<0.005, **p<0.01 and ***p<0.001 by Student’s t-test.

Increasing evidence indicates that the intratumoral heterogeneity is a critical factor that leads to chemoresistance.^32^ We speculate that the tumor heterogeneity caused different response rates to chemotherapeutics, which may also account for the failure of ICB therapy.^33 34^ To investigate whether NSCLC cells respond to pemetrexed treatment differently and generate drug-resistant subpopulations, we selected CL1-5-derived subclones that displayed different pemetrexed responses and classified them into two groups, PEM-R and non-responder (PEM-NR) (figure 4A, B). Interestingly, we found that the PEM-NR cell clones expressed extremely low levels of PD-L1 at both the mRNA and protein levels, and had little response, if any, to the pemetrexed-induced PD-L1 upregulation (figure 4A, B). This suggests that pemetrexed might fail to stimulate certain signaling(s) in this subpopulation that lost the ability to drive the transcriptional activation of *PD-L1* gene (ie, *CD274*). To dissect the molecular mechanisms by which pemetrexed may render distinct regulations on *PD-L1* expression depending on different cellular contexts, we performed RNA-sequencing (RNA-seq) for each CL1-5 subclone (PEM-R1, R2, NR1, or NR2) and compared transcriptome profiles between the PEM-R and PEM-NR groups. An estimate of 337 genes, which matched our criteria for upregulation in the PEM-R group and downregulation in the PEM-NR group, were identified (online supplemental figure S4A). Gene ontology enrichment analysis further identified that the NF-κB regulatory pathway and its related genes have significant changes in their expressions in response to pemetrexed treatment (figure 4C, D). This encouraged us to investigate the potential regulation of pemetrexed in the NF-κB signaling pathway.

NF-κB activation is tightly controlled by IKK-mediated phosphorylation of IκB, which normally sequestrates NF-κB in the cytosol.^35^ On the phosphorylation by IKK, IκB is degraded through proteasome-mediated mechanisms, thereby releasing NF-κB and enabling it to enter the nucleus for its transactivation function.^35 36^ We first investigated the effects of pemetrexed on NF-κB signaling by monitoring the phosphorylation status of IκB-α. Interestingly, we observed that the phosphorylated IκB-α can only be detected in pemetrexed-responsive CL1-5 subclones but not in non-responsive subclones (figure 4E and online supplemental figure S4B). The pemetrexed-induced IκB-α phosphorylation was accompanied by a significant loss of IκB-α protein and the phosphorylation of NF-κB p65; both events facilitate NF-κB to enter the nucleus and bind to the promoters of target genes.^37^ These data strongly suggest that pemetrexed triggers the activation of NF-κB signaling, and thereby transactivates *PD-L1* gene expression. Supporting the idea, luciferase reporter experiments in CL1-5 subclones demonstrated that pemetrexed induced the transactivation activity of NF-κB in a dose-dependent manner, which is only detectable in pemetrexed-responsive CL1-5 subclones (figure 4F and online supplemental figure S4C). The mRNA expression levels of *PD-L1* in each group of CL1-5 subclones also displayed the same patterns with corresponding protein expression levels when cells responded to pemetrexed treatment (figure 4G and online supplemental figure S4D). To further confirm that the NF-κB signaling pathway acts downstream of pemetrexed to transactivate *PD-L1* expression, we treated the pemetrexed-responsive CL1-5 subclones with an IKK inhibitor, BAY11-7082, to suppress the phosphorylation of IκB-α. As expected, the treatment of BAY11-7082 significantly repressed the pemetrexed-induced PD-L1 expression at both the mRNA and protein levels (figure 4H, J and online supplemental figure S4E). Moreover, silencing the major subunit of NF-κB p65 (encoded by *RelA*) by siRNA oligonucleotides (siRelA) could also recapitulate the effects of BAY11-7082 on the pemetrexed-responsive CL1-5 subclones; as we found that the knockdown of p65 greatly suppressed the ability of pemetrexed to induce PD-L1 upregulation (figure 4I, K and online supplemental figure S4F). Taken together, we demonstrated that pemetrexed induces *PD-L1* expression through the engagement of NF-κB-mediated transactivation activity in NSCLC cells.

### Pemetrexed induces *PD-L1* gene transcription through the TS−ROS−NF-κB regulatory axis

**Figure 5:**
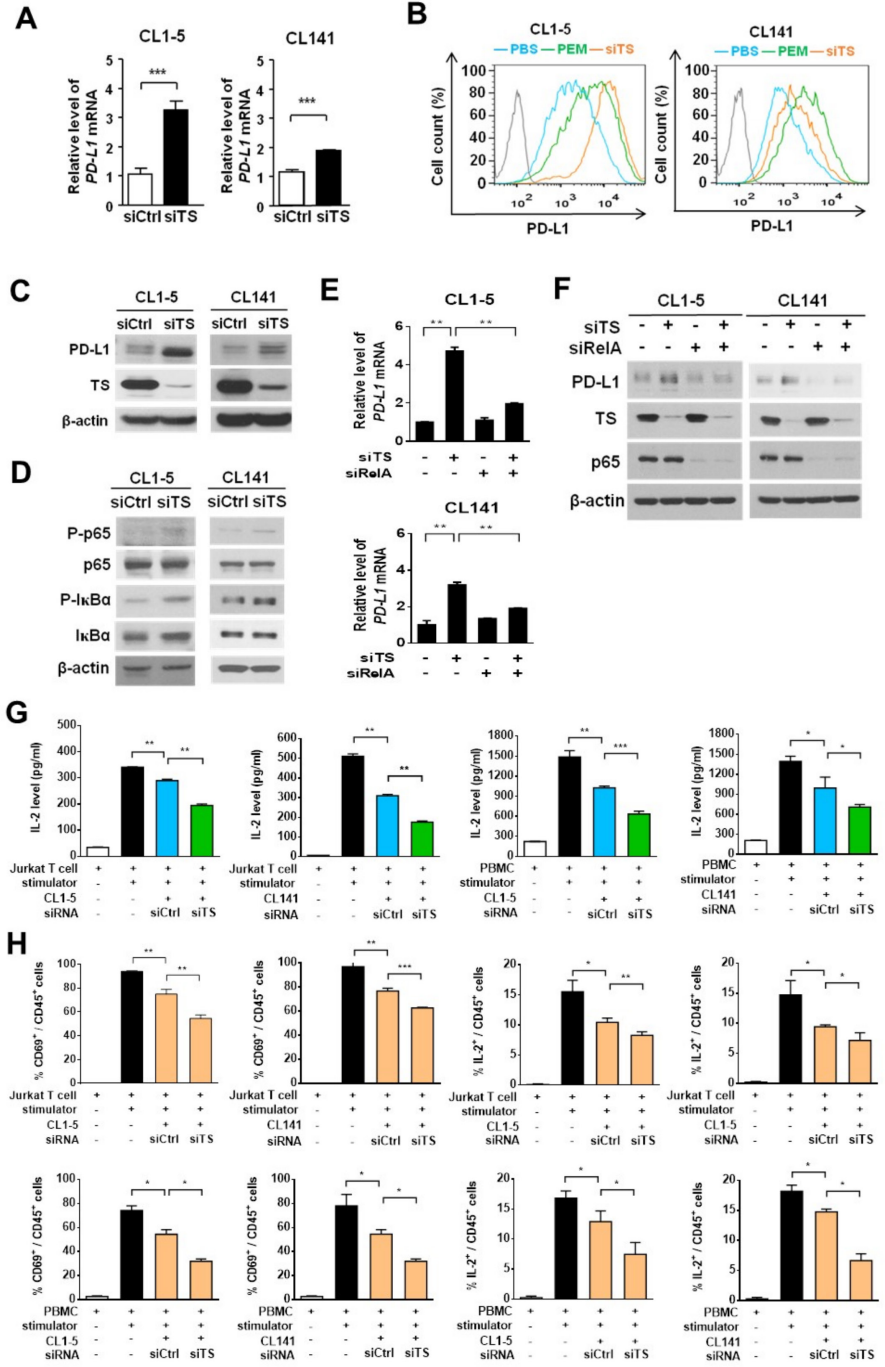
Knockdown of thymidylate synthase (TS) induces programmed death-ligand 1 (PD-L1) expression in non-small-cell lung cancer (NSCLC) cells and decreases the production of interleukin-2 (IL-2) by activated T cells in the NSCLC and T cell coculture system. (A–D), CL1-5 or CL141 cells transfected with control-siRNA (siCtrl) or *TS*-siRNA (siTS) oligonucleotides were lysed and analyzed by qRT-PCR (A), flow cytometry (B), or immunoblotting (C, D) 72 hours after transfection. Data are shown as means and SD for three independent experiments (n=3). (E, F) CL1-5 or CL141 cells transfected with control-siRNA (siCtrl), TS-siRNA (siTS) and RelA-siRNA (siRelA) oligonucleotides in different combination were lysed and analyzed by qRT-PCR (E) or immunoblotting (F), 72 hours after transfection. (G, H) CL1-5 or CL141 cells were transfected with siCtrl or siTS siRNA oligonucleotides for 24 hours and followed by cocultured with Jurkat T-cells or PBMCs at different cancer to T cell ratios in the presence of the 1×T cell stimulation cocktail for additional 48 hours. (G) IL-2 levels were measured by ELISA. (H) The levels of CD69 and intracellular IL-2 produced by Jurkat T-cells or PBMCs were measured by flow cytometry. **P<0.01 and ***p<0.001 by Student’s t test.

We next sought to trace the direct target(s) of pemetrexed that is required for its PD-L1 priming activity. Since the JAK1/2 kinase family has been reported to mediate PD-L1 expression on extracellular stimulation in tumor cells,^38^ we first investigated the effects of ruxolitinib (a JAK inhibitor) on the expression of PD-L1 in pemetrexed-treated CL1-5 and CL141 cells. Our results showed that ruxolitinib did not have any effect on pemetrexed-dependent upregulation of PD-L1 (online supplemental figure S5), suggesting that pemetrexed might regulate PD-L1 expression through a different target(s). It is also known that pemetrexed and 5-FU can inhibit DNA biosynthesis processes by suppressing the activity of TS, an enzyme that catalyzes the transformation of deoxyuridine monophosphate to deoxythymidine monophosphate for the maintenance of DNA replication and repair, thereby inducing DNA damage in tumor cells through TS inhibition.^17 39^ Although there is no evidence showing TS being involved in gene regulation, we tried to evaluate the possibility that TS might be the direct target of pemetrexed for regulating *PD-L1* gene expression. We performed RNAi silencing approaches to knock down the endogenous *TS* expression in CL1-5 and CL141 cells. We found that both mRNA and protein expression of PD-L1 significantly elevated on the knockdown of TS (figure 5A, C), suggesting that TS is capable of regulating *PD-L1* gene expression. Essentially, knockdown of TS greatly elevated membrane-bound PD-L1 levels in CL1-5 and CL141 cells (figure 5B), thereby enhancing the ability of tumor cells to suppress Jurkat T-cell or CTL activation (figure 5G). Thus, these results suggest that pemetrexed may upregulate *PD-L1* expression through the inactivation of the TS-dependent DNA synthesis pathway. The elevated PD-L1 levels on the cell surface of NSCLC cells, in turn, diminish immune responses by suppressing T-cell activation (eg, IL-2 secretion).

Since we identified that the conventional pemetrexed target TS as well as the NF-κB signaling, both involved in *PD-L1* gene expression, we explored whether the TS-dependent DNA synthesis pathway can crosstalk with the NF-κB signaling pathway. Knockdown of TS significantly induced the phosphorylation of IκB-α and NF-κB p65 in CL1-5 and CL141 cells, indicating that TS is capable of cross-regulating the NF-κB activity (figure 5D). To further confirm this idea and verify whether TS acts upstream of the NF-κB pathway to regulate *PD-L1* expression, we cosilenced the expression of *TS* and *RelA* by specific siRNA oligonucleotides in CL1-5 and CL141 cells. We found that knockdown of TS by *TS*-siRNA induced PD-L1 upregulation at both the mRNA and protein levels, while the effects of *TS*-siRNA on PD-L1 expression were abolished by the treatment of *RelA*-siRNA (figure 5E, F). This confirms that the NF-κB pathway acts downstream of TS for the control of PD-L1 expression.

**Figure 6:**
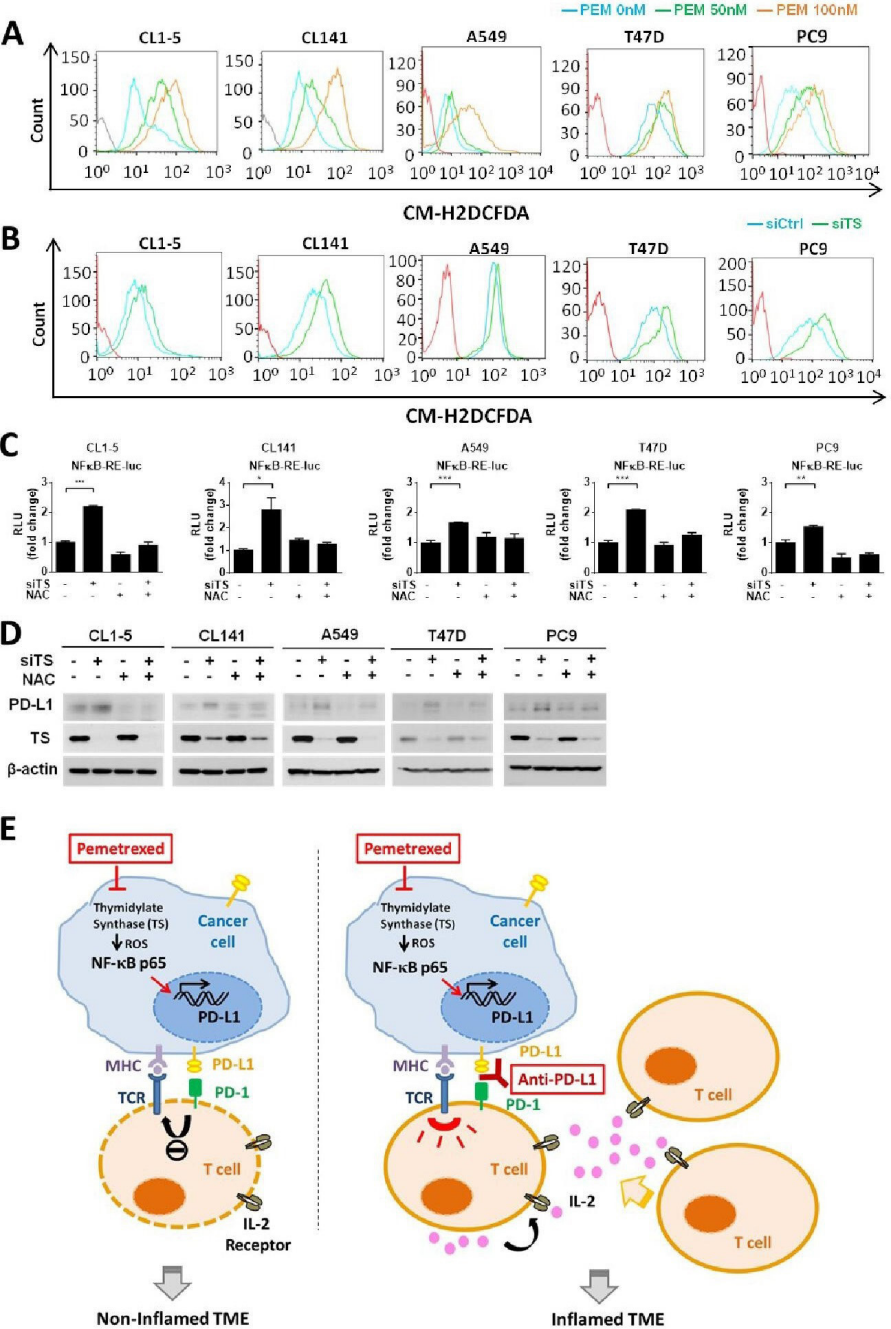
The thymidylate synthase (TS)−reactive oxygen species (ROS)−NF-κB regulatory axis mediates pemetrexed (PEM)-induced programmed death-ligand 1 (PD-L1) upregulation. (A, B) CL1-5, CL141, A549, T47D or PC9 cells were treated with PEM (50 or 100 nM) alone or in combination with control siRNA (siCtrl) or *TS*-siRNA (siTS) for 24 hours, followed by incubation with the DCFDA fluorogenic dye (10 µM) for 30 min. The levels of ROS were assessed by flow cytometry. (C) CL1-5, CL141, A549, T47D or PC9 cells transfected with the NF-κB−Luc reporter plasmid in combination with control siRNA (siCtrl) or *TS*-siRNA (siTS) were treated with N-acetylcysteine (NAC) (5 mM) or left untreated, for 72 hours. The luminescence signal from the NF-κB–Luc reporter (firefly luciferase) was normalized to that of cotransfected pRL-TK vector (Renilla luciferase) to control for transfection efficiency and was expressed as relative light units. Data are shown as means and SD for three independent experiments (n=3). (D) CL1-5, CL141, A549, T47D or PC9 cells transfected with control siRNA (siCtrl) or *TS*-siRNA (siTS) were treated with NAC (5 mM) or left untreated, for 72 hours. Cells were lysed and analyzed by immunoblotting with the indicated antibodies. (E) Proposed model illustrating the molecular basis of the combinational treatment of PEM and anti-PD-L1 antibody in non-small-cell lung cancer (NSCLC). PEM induces the inhibition of TS, which in turn increases intracellular levels of ROS and results in the activation of NF-κB signaling pathway. NF-κB then transactivates *PD-L1* expression and increases the levels PD-L1 on the cell surface of tumor cells. The upregulation of PD-L1 expression in tumor cells may suppress T-cell activation through the PD-L1/PD-1 engagement and lead to non-T-cell-inflamed tumor microenvironment (left). However, the combinational treatment of PEM and anti-PD-1/PD-L1 antibodies reactivates T-cell activities and improves effector function (right). MHC, major histocompatibility complex; TCR, T-cell receptor; IL-2, Interleukin-2; TME, tumor microenvironment.

High level of ROS in tumor cells has been connected to the activation of NF-κB signaling in tumor inflammation.^40–42^ The intracellular ROS may enhance the phosphorylation of IκB and lead to the proteasomal degradation of IκB, which in turn induces the release and translocation of NF-κB to the nucleus to initiate transcription. Interestingly, the inhibition of TS by TS inhibitors has also been shown to increase the intracellular level of ROS.^43^ To explore whether pemetrexed-mediated TS inhibition could increase the intracellular level of ROS and result in the activation of NF-κB signaling pathway, we detected intracellular levels of ROS in CL1-5 and CL141 cells on the inhibition of TS. As expected, we found that intracellular levels of ROS were significantly elevated on inhibition of TS by either pemetrexed or TS knockdown (figure 6A, B). Luciferase reporter experiments in CL1-5 and CL141 cells further demonstrated that knockdown of TS induced the transactivation activity of NF-κB, while the effects of TS-knockdown on NF-κB could be rescued when the intracellular ROS was effectively scavenged by the antioxidant, N-acetylcysteine (NAC) (figure 6C). Additionally, we demonstrated that 5-FU, an antimetabolic chemotherapeutic that is also known to target TS and inhibit DNA biosynthesis, has the potential to increase the intracellular level of ROS and result in the activation of NF-κB signaling pathway in NSCLC cells (online supplemental figure 6A-C). These data indicate that antimetabolites, pemetrexed and 5-FU, can induce the activation of NF-κB signaling by regulating the activity of TS and the intracellular level of ROS. To further confirm that this TS−ROS−NF-κB regulatory axis contributes to the upregulation of PD-L1 in NSCLC cells, we checked the PD-L1 level in this scenario. Indeed, knockdown of TS promoted PD-L1 upregulation in CL1-5 and CL141 cells, while the effects can be abolished if the intracellular ROS is reduced effectively by NAC (figure 6D). To further confirm that the TS−ROS−NF-kB regulatory axis is actively involved in the activation of *PD-L1* transcription in tumor cells, we selected A549, PC9 human lung cancer and T47D human breast cancer cell lines that are known not to express PD-L1^44 45^ (online supplemental figure S7A), and tested the effects of TS inhibition on these tumor cells by either pemetrexed or TS knockdown. We found that pemetrexed greatly induced the intracellular level of ROS in A549, PC9 and T47D cells (figure 6A). Consistently, knockdown of TS induced the intracellular level of ROS, the transactivation activity of NF-κB, as well as PD-L1 levels in these cancer cell lines while the effects of TS inhibition could be significantly abolished by the antioxidant NAC (figure 6B–D and online supplemental figure S7B). These results strongly support a general function of the TS−ROS−NF-κB regulatory axis in transcriptional regulation of *PD-L1* in tumor cells.

In conclusion, our results indicate that pemetrexed can induce PD-L1 upregulation in human NSCLC cells by regulating the TS−ROS−NF-κB regulatory axis and prime a favorable tumor microenvironment for ICB therapy (figure 6E).

## Discussion

The folate antimetabolite pemetrexed is one of the most commonly used chemotherapeutics in NSCLC treatment. Recently, the combination of pemetrexed-based chemotherapy and ICB therapy has demonstrated persuasive clinical activity in patients with NSCLC with cancer metastasis. These clinical results led to the initial accelerated approval of this chemoimmunotherapy combination for patients with advanced NSCLC.^19 20^ Although this novel combinatory therapy has become first-line treatment in advanced NSCLC, very little is known about the underlying antitumor mechanisms of pemetrexed in combination with immunotherapy. In the present study, we show that pemetrexed, but not the other commonly used chemotherapeutics (eg, cisplatin and PTX), is capable of inducing PD-L1 expression in NSCLC cells that further primes the tumor microenvironment favorable to the ICB therapy. We find that pemetrexed and anti-PD-1/PD-L1 antibody synergistically induce T-cell activation in vitro and in vivo, mainly through restoring the exhausted T-cell activities. Additionally, we reveal a novel molecular mechanism of how pemetrexed induces PD-L1 upregulation in tumor cells. Pemetrexed suppresses the activity of TS and in turn increases the intracellular level of ROS, which further triggers the NF-κB signaling pathway and the following activation of *PD-L1* gene transcription in NSCLC cells. Interestingly, pemetrexed fails to trigger *PD-L1* transcriptional activation in a subpopulation of NSCLC cells where the NF-κB signaling pathway is unregulated. Thus, the role of pemetrexed in the aforementioned chemoimmunotherapy may need to be reinterpreted by the thought that pemetrexed can exert immunomodulatory effects through multiple pathways (discussed next). Furthermore, the differential responses of tumor cells from individual patients to the pemetrexed-induced PD-L1 expression may need to be analyzed for the pemetrexed-based immunotherapy.

It is now clear that tumor cells escape immune surveillance and attack by upregulating the expression of PD-L1, which can interact with the immunosuppressive molecule PD-1 on T cells to inhibit the function of T cells and induce T-cell exhaustion.^11 46^ Through the inhibition of PD-1/PD-L1 interaction, monoclonal antibodies targeting PD-1 or PD-L1 have shown potent efficacy in reducing immunosuppressive signals within the tumor microenvironment and in increasing T cell-mediated antitumor immunity.^47^ Here, we demonstrate that the beneficial effects of pemetrexed in the FDA approval chemoimmunotherapy^19–21^ are mediated, at least in part, by its ability to upregulate the expression of PD-L1 in NSCLC cells and synergize with PD-1/PD-L1 blockade to restore exhausted T-cell activities. TILs, CD4^+^ and CD8^+^ T cells, can secrete antitumor cytokines such as IL-2, TNF-α and IFN-γ, while the effector activities for producing these cytokines could be extinguished due to T-cell exhaustion.^30 31^ Our data from the NSCLC/T cell coculture systems and syngeneic mouse models indicate that pemetrexed alone suppresses the ability of T cells to produce IL-2 and IFN-γ because of PD-L1 upregulation, while the levels of IL-2 and IFN-γ can be restored or even further enhanced in the combinational treatment of pemetrexed and anti-PD-1/PD-L1 antibodies. We also demonstrate that pemetrexed can synergize with PD-1/PD-L1 blockade to enhance the production of IL-2, TNF-α and IFN-γ within the tumor microenvironment in the mouse syngeneic models. Interestingly, pemetrexed alone is capable of inducing the production of IL-2, TNF-α and IFN-γ in the tumor area, suggesting that the cytotoxic effects of pemetrexed on tumor cells may also activate antigen-presenting cells (APCs), such as macrophages and dendritic cells, or other effector cells, such as natural killer cells, that are known to produce TNF-α and IFN-γ.^48 49^ More interestingly, our data indicates that platinum-based chemotherapy, for example, cisplatin, cannot induce the expression of PD-L1 in NSCLC cells and provides no advantage in the combinatory therapy of pemetrexed and anti-PD-L1 antibody for lung cancer treatment. This strongly suggests that pemetrexed can possibly be combined with ICB therapy without platinum-based chemotherapy. Further clinical investigations are therefore needed to test the potential of pemetrexed alone in combination with ICB therapy.

Although we show that pemetrexed primes a favorable tumor microenvironment for ICB therapy via inducing PD-L1 expression in tumor cells, another possible mechanism can be attributed to the cytotoxic effects of pemetrexed in inducing immunogenic cell death (ICD). ICD induces the chronic exposure of immune-stimulating factors from dying tumor cells, which activates APCs to induce T-cell priming and antitumor adaptive immunity.^24 25^ Indeed, Schaer *et al* recently reported that pemetrexed monotherapy resulted in upregulation of multiple immune-related genes and increased T-cell activation in mouse MC38 and Colon26 colorectal tumor models via inducing ICD in mouse tumor cells.^26^ Consistent with previous observations, our data show that pemetrexed induces significant increase in the number of CD4^+^ cells infiltrating the tumors in mouse CT26 colorectal and LL2 lung tumor models. Additionally, our RNA-seq data finds the level of MHC class II genes, for example, HLA-DQB1 and HLA-DMA, being upregulated in pemetrexed- responsive CL1-5 subclones (data not shown). Thus, our findings indicate that pemetrexed could also induce ICD and trigger CD4^+^ T helper cells to infiltrate tumors. The ICD induction could also be attributed to the pemetrexed-induced ROS production, which is known to target the endoplasmic reticulum (ER) and induce ER stress.^50^ More importantly, we find that the pemetrexed-induced ROS production triggers NF-κB signaling and induces the transcriptional activation of *PD-L1* gene. Our findings therefore suggest that pemetrexed may exert a dual role in facilitating ICB therapy; pemetrexed can induce ICD to activate APCs and trigger the tumor infiltration of CD4^+^ T helper cells, and also induce the expression of PD-L1 in tumor cells that boosts the effects of ICB on the activation of CD8^+^ cytotoxic T cells.

Several studies indicate that high expression of the conventional pemetrexed target, TS, is associated with pemetrexed resistance.^51–53^ However, these observations cannot fully explain the phenomena observed in clinical NSCLC samples,^54^ raising the possibility that other mechanism(s) might exist, which can be responsible for pemetrexed resistance. We demonstrate here that the NF-κB signaling pathway plays an essential role in the transcriptional activation of *PD-L1* in NSCLC cells, and such signaling regulation can be triggered by the pemetrexed-induced ROS production. Interestingly, we also identify a subpopulation of lung cancer cells that does not respond to the pemetrexed-induced PD-L1 upregulation with its NF-κB signaling pathway unregulated. The unregulated NF-κB signaling might be due to the dysfunction of IKK, an upstream kinase for the phosphorylation of IκB and the subsequent proteasomal degradation, in such lung cancer subpopulation where the IκB level is relatively high and the phosphorylation of IκB cannot be induced on pemetrexed stimulation (figure 4E and online supplemental figure S4B). Thus, the potential role of IKK or other upstream regulators of NF-κB signaling in the pemetrexed-resistance mechanism may be considered and warrants further investigation.

Although the pemetrexed-induced ROS accumulation might also cause cellular DNA damage and affect the stages of cell cycle, we found that there is no significant change in the cell stages of NSCLC cells (eg, H1299, CL141, CL1-5 parental, R1, R2, NR1, and NR2) in response to pemetrexed (online supplemental figure S8A). Our data indicate that the sublethal dose (50 or 100 nM) of pemetrexed may only cause the induction of *PD-L1* transcription via the TS−ROS−NF-κB regulatory axis but not affect cell stages, such as cell apoptosis and cell cycle arrest. Interestingly, we found that the stages of the cell cycle in pemetrexed non-responsive CL1-5 subclones (PEM-NR1, NR2) are different to that in pemetrexed-responsive subclones (PEM-R1, R2). Our results showed an increased percentage of cells in G1 phase and a decreased percentage of cells in S phase in non-responsive subclones (NR1, NR2) as compared with the responsive subclones (R1, R2) (online supplemental figure S8B). It is consistent with previous findings that NF-kB is capable of promoting G1/S transition while overexpression of IκB-α or inactivation of NF-kB causes a retarded G1/S transition.^55 56^ Although we found that cell cycle regulation of the pemetrexed-responsive and non-responsive NSCLC subpopulations may be different due to differential NF-kB activities, whether pemetrexed effects differentially to a specific cell stage of NSCLC might warrant further investigation.

The roles of NF-κB signaling in cancer have been linked to cancer progression via its function in preventing apoptosis and enhancing cell proliferation.^57 58^ Our data reveal that antimetabolites, pemetrexed and 5-FU, are capable of inducing the transcriptional activation of *PD-L1* via the TS−ROS−NF-κB regulatory axis in NSCLC cells, providing an additional linkage between NF-κB signaling and chemotherapy-induced immunosuppression. Consistent with our findings, Peng *et al* reported that PTX and gemcitabine can upregulate *PD-L1* gene expression in ovarian cancer cells via increasing cellular NF-κB p65 protein, which fosters consequently an immunosuppressive tumor microenvironment in ovarian cancer.^59^ Together our results indicate that NF-κB can exert its transactivation function to activate *PD-L1* gene expression in cancer cells, while the mechanism of action of chemotherapeutic agents in the induction of NF-κB signaling could be variant and dependent on specific cellular contexts.

Our findings provide new insights into the molecular mechanism by which pemetrexed induces PD-L1 upregulation in tumor cells through the TS−ROS−NF-κB regulatory axis. It also offers an indication to avoid unnecessary treatments for lung cancer patients due to cisplatin providing no advantage in the combinatory therapy of pemetrexed and anti-PD-L1 antibody.

## Conclusions

In this study, we found that pemetrexed, instead of the platinum analog cisplatin, can improve PD-L1 expression in advanced NSCLC cells through the inactivation of TS follow by the accumulation of intracellular ROSs and activation of the NF-κB pathway. By using syngeneic tumor models, we confirmed that the combinatory treatment of pemetrexed (without cisplatin) with PD-1/PD-L1 blockage antibodies is sufficient to inhibit tumor growth through the activation and/or recruitment of tumor-infiltrating CD4 and CD8 T-lymphocytes. Our results provide fundamental insights into the regulatory mechanism of pemetrexed in the improvement of ICB therapy in lung cancer treatment.
