# Supplementary material for: Antimetabolite pemetrexed primes a favorable tumor microenvironment for immune checkpoint blockade therapy
Source: J Immunother Cancer. 2020 Nov 26;8(2):e001392. doi: 10.1136/jitc-2020-001392 (PMC7692992; doi:10.1136/jitc-2020-001392)
Supplement: Supplementary data [file jitc-2020-001392supp001.pdf]

Suppl. Table S1. IC_{50} values of various chemotherapeutic agents in cancer cell lines.

	CL1-5	CL1-5 _R1	CL1-5 _R2	CL1-5 _NR1	CL1-5 _NR2	CL141	H1299	CT26	LL2
**Pemetrexed (PEM) (nM)**	280.7	139.6	182	359.1	378.3	636.5	1070	>5uM	173.9
**Fluorouracil (5-FU) (μM)**	1.2	1.89	1.43	1.03	4.378	8.1	12.2	25.46	7.5
**Paclitaxel (PTX) (nM)**	3.2	9.86	19.47	23.14	6.01	3.5	17.3	51.69	56.29
**Cisplatin (μM)**	18.4	8.7	11	8.15	7.06	12.8	21.9	7.86	3.16

Indicated cancer cell lines (5×10^3 cells/well) were seeded in 96-well plates overnight and the culture medium was replaced with fresh medium containing drugs at concentrations of 0.001–10 μM. After 72 hours, cell viability was assessed with the SRB assay to determine IC_{50} values. Values shown are the mean ± SD from three independent experiments.
