# Supplementary material for: Antimetabolite pemetrexed primes a favorable tumor microenvironment for immune checkpoint blockade therapy
Source: J Immunother Cancer. 2020 Nov 26;8(2):e001392. doi: 10.1136/jitc-2020-001392 (PMC7692992; doi:10.1136/jitc-2020-001392)
Supplement: Supplementary data [file jitc-2020-001392supp002.pdf]

Supplementary Figures

Suppl. Figure S1

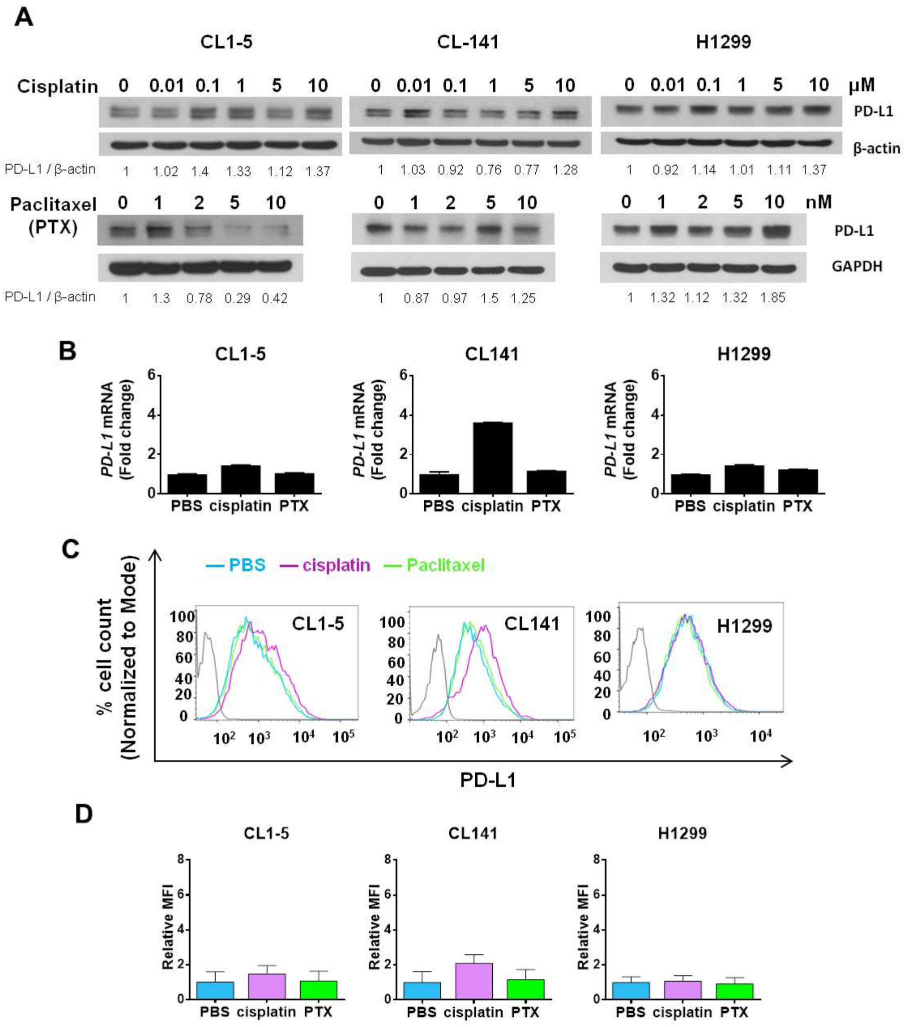

Figure S1.

Cisplatin and paclitaxel (PTX) did not upregulate PD-L1 expression in NSCLC cell lines. (A) Detection of PD-L1 in NSCLC cell lines CL1-5, CL141 and H1299 treated with sub-lethal concentrations of cisplatin, PTX or the vehicle control (PBS) for 72 hours by immunoblotting**.** The expression of β-actin serves as a loading control. The

ratios between the intensity of the bands corresponding to PD-L1 and those corresponding to β-actin were calculated. (B-D) CL1-5, CL141 and H1299 cells were treated with PBS, 10 μM cisplatin, or 5 nM PTX for 72 hours. Relative *PD-L1* mRNA expression levels, determined by qRT-PCR, are shown in B. Representative histograms for PD-L1 expression levels on the cell surface of NSCLC cells obtained by flow cytometry are shown in C. Relative mean fluorescence intensity (MFI) for PD-L1 expression levels is shown in D. All the data are shown as means and s.e.m. for three independent experiments (n = 3).

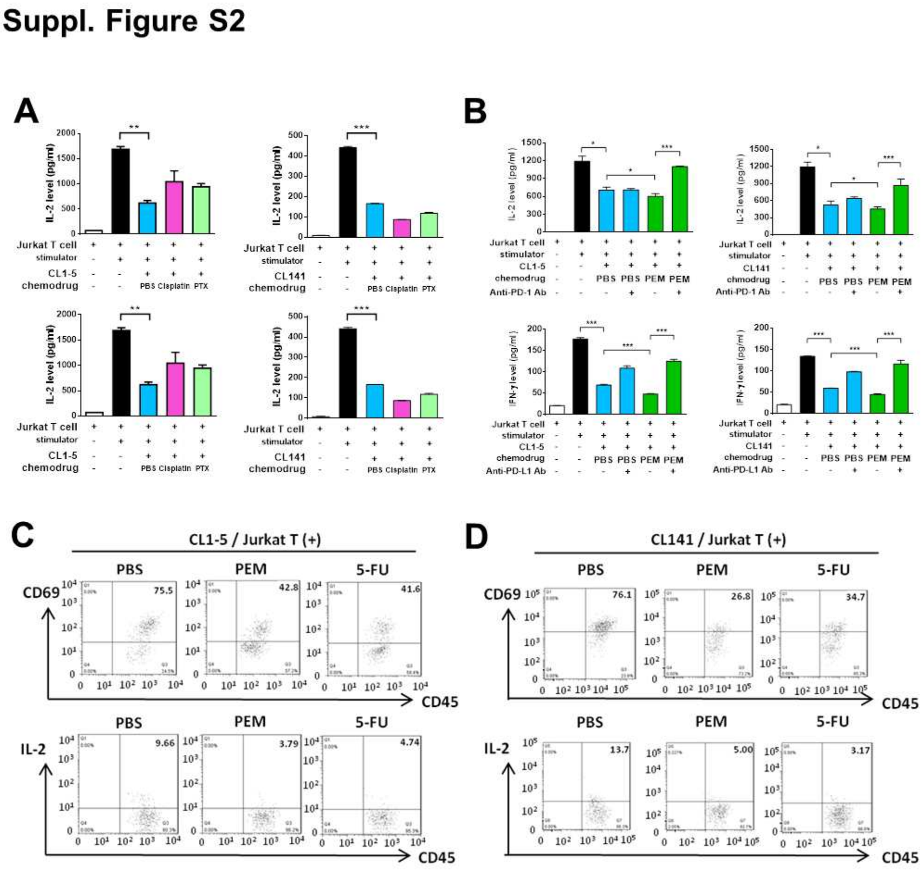

Figure S2.

(A) Cisplatin and paclitaxel (PTX) did not suppress IL-2 production by activated T cells in the NSCLC and T cell coculture system. CL1-5 or CL141 cells were pre-incubated with 10 μM cisplatin, 5 nM PTX or the vehicle control (PBS) for 48 hours, and subsequently co-cultured with activated Jurkat T cells or PBMC cells at different cancer to T cell ratios in the presence of 1 x T cell stimulation cocktail for additional 48 hours. (B) CL1-5 or CL141 cells were pre-incubated with 100 nM pemetrexed (PEM) or the vehicle control (PBS) for 48 hours, and subsequently co-cultured with activated Jurkat T-cells in the presence of 1 x T cell stimulation

cocktail with or without anti-PD-1 antibody (10 ng/ml) or anti-PD-L1 antibody (10 ug/ml) for additional 48 hours. The levels of IL-2 and IFN-γ were measured by ELISA. Data are shown as means and s.d. for three independent experiments (n = 3). (C, D) CL1-5 or CL141 cells were pre-incubated with 100 nM PEM, 5 μM 5-fluorouracil (5-FU) or the vehicle control (PBS) for 48 hours, and subsequently co-cultured with activated Jurkat T-cells in the presence of 1 x T cell stimulation cocktail for additional 48 hours. The intracellular levels of CD69 and IL-2 produced by Jurkat T-cells were measured by flow cytometry.

Lu C-S, *et al. J Immunother Cancer* 2020; 8:e001392. doi: 10.1136/jitc-2020-001392

Suppl. Figure S3

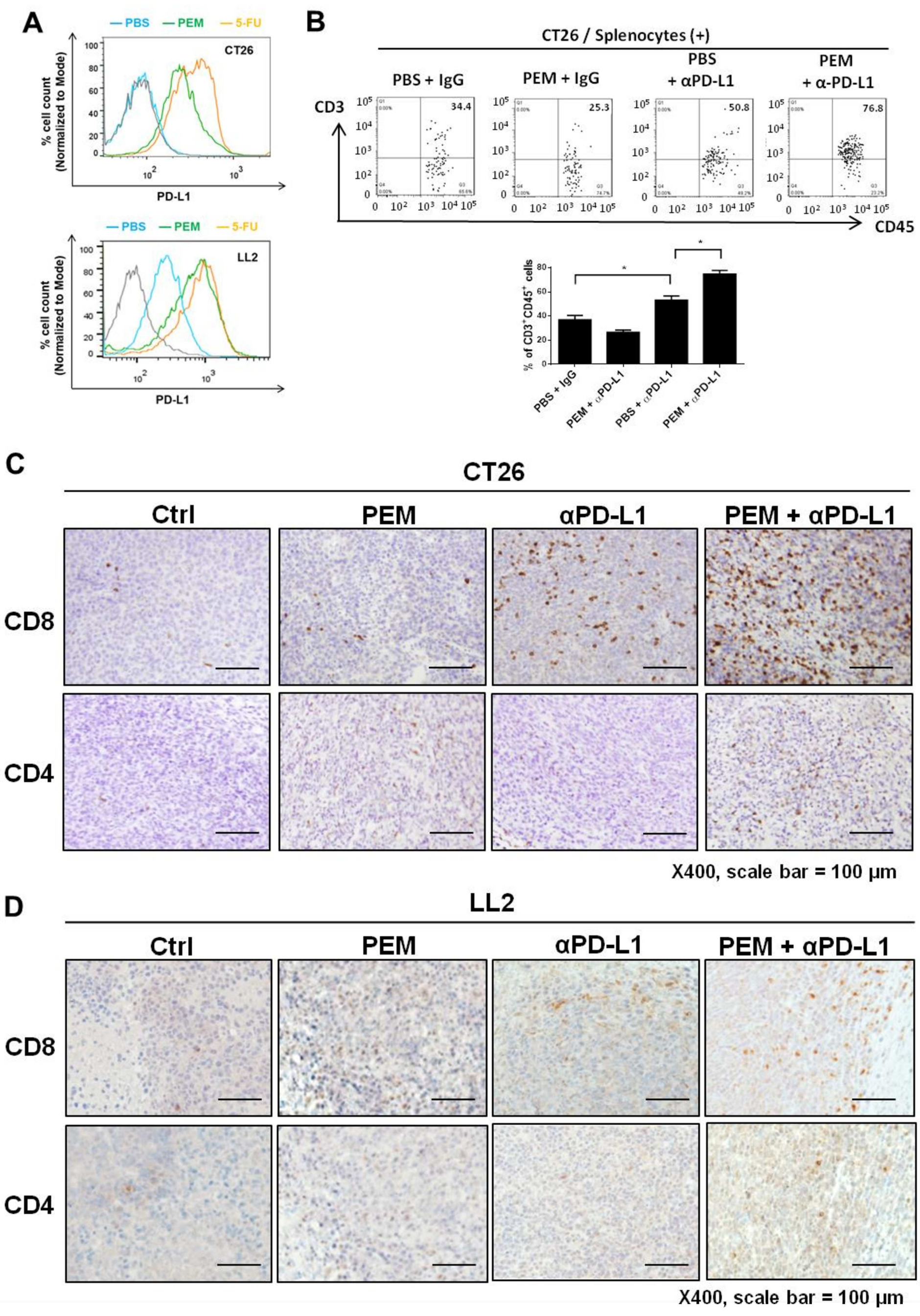

Lu C-S, et al. J Immunother Cancer 2020; 8:e001392. doi: 10.1136/jitc-2020-001392

Figure S3.

Pemetrexed induces PD-L1 expression in CT26 and LL2 cells and synergizes with anti-PD-L1 antibody to activate $CD4^{+}$ and $CD8^{+}$ T cells in mouse syngeneic tumor models. (A) Murine CT26 or LL2 cells were treated with 100 nM Pemetrexed (PEM), 5 μM 5-fluorouracil (5-FU) or the vehicle control (PBS) for 72 hours. Cells were then incubated with anti-PD-L1-PE antibody or the IgG-PE isotype for the detection of the membrane-bound PD-L1 by flow cytometry. The mean fluorescent intensity (MFI) obtained by flow cytometry indicates the expression levels of PD-L1 on the cell surface. (B) Spleen was removed from wild mouse immediately after killing; erythrocytes were lysed, and spleen-derived lymphocytes from each mouse were then resuspended in RPMI 1640 with 10% heat-inactivated FBS. CT26 cells were pre-incubated with 100 nM PEM or the vehicle control (PBS) for 48 hours, and subsequently co-cultured with activated spleen-derived lymphocytes (splenocytes) in the presence of 1 x T cell stimulation cocktail with or without anti-PD-L1 antibody (10 μg/ml) for additional 48 h. Representative dot plots for the indicated cell percentages determined by flow cytometry. Quantitative plots indicating the CD3 staining in $CD45^{+}$ lymphocytes. (C, D) Immunohistochemical staining of CD4 and CD8 in serial sections of tumor specimens in the CT26 (C) and LL2 (D) tumor models.

Lu C-S, *et al. J Immunother Cancer* 2020; 8:e001392. doi: 10.1136/jitc-2020-001392

Suppl. Figure S4

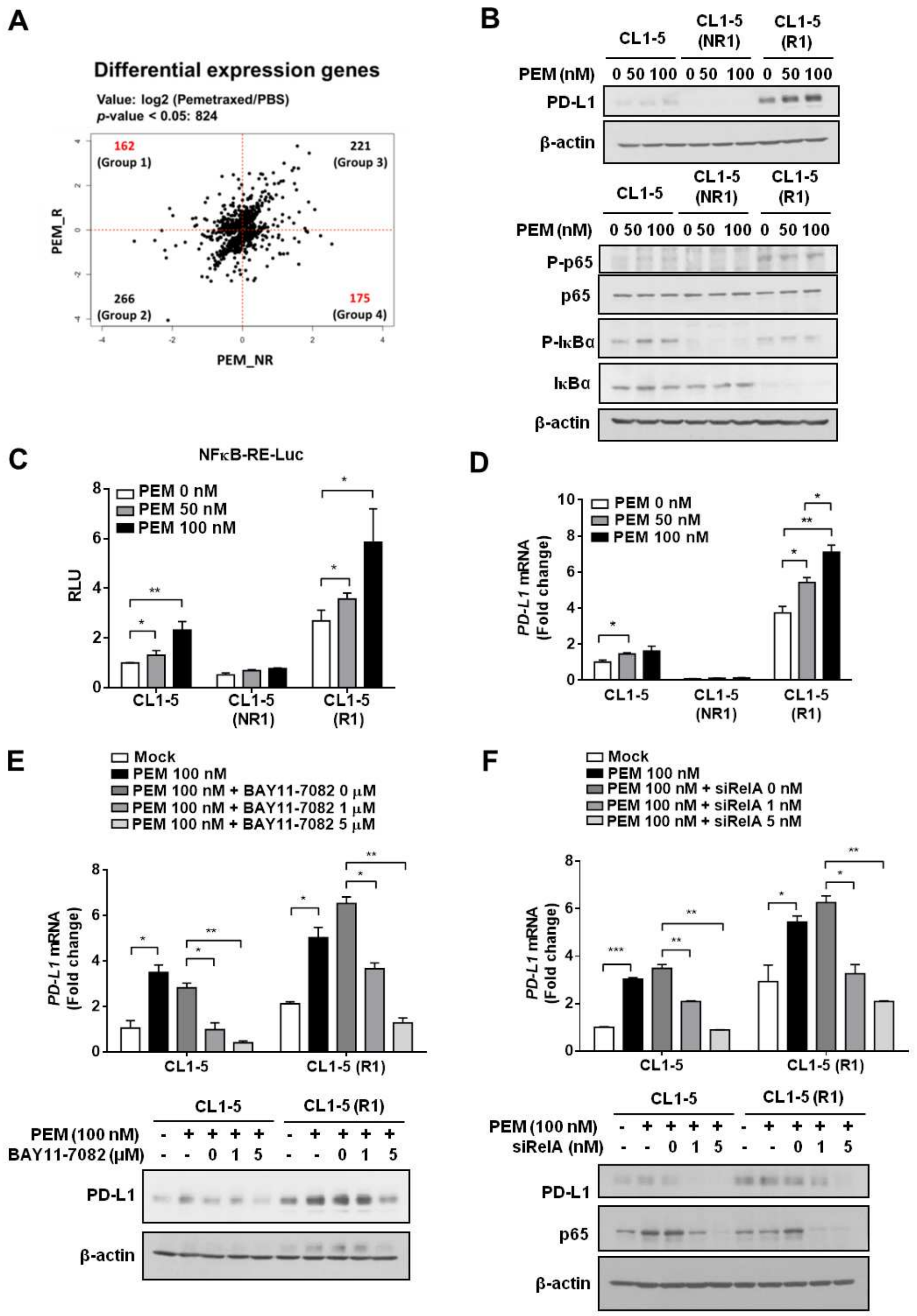

Figure S4.

NF-κB signaling acts downstream of pemetrexed (PEM) to induce the transcriptional activation of *PD-L1* in NSCLC cells. (A) Scatterplot showing differential expression genes from two CL1-5 subclone groups, PEM_NR and PEM_R, when responded to PEM treatment. The X and Y coordinates represent log2 fold changes in gene expression of individual genes in CL1-5 subclones, PEM_NR and PEM_R, respectively. (B) CL1-5, CL1-5/NR1, and CL1-5/R1 cells were treated with 50 or 100 nM PEM or the vehicle control (PBS) for 72 hours. The expression of the indicated total or phosphorylated proteins was evaluated by immunoblotting. The levels of β-actin serve as a loading control. (C) Transcriptional activation of a NF-κB reporter gene in PEM-treated parental and subclonal CL1-5 cells. NF-κB–Luc, a NF-κB responsive promoter–reporter construct, was transfected into each CL1-5 cell clone as indicated. The luminescence signal from the NF-κB–Luc reporter (firefly luciferase) was normalized to that of co-transfected pRL-TK vector (Renilla luciferase) to control for transfection efficiency and was expressed as relative light units (RLU). Data are shown as means and s.d. for three independent experiments (n = 3). (D) Relative mRNA expression levels of *PD-L1*, determined by qRT-PCR, in each CL1-5 cell clone in response to PEM. (E) Inhibition of NF-κB signaling impairs the PEM-induced PD-L1 upregulation. Each CL1-5 cell clone was co-treated with PEM (100 nM) and different doses of the IKK inhibitor (BAY117082) for 3 days. Total RNA was isolated and relative *PD-L1* mRNA levels were determined by qRT-PCR. The same experiment was performed and the protein levels of PD-L1 were evaluated with immunoblotting (bottom). (F) Knockdown of NF-κB p65 reverses the effects of PEM on the expression of PD-L1. CL1-5 or CL1-5/PEM-R1 cells were transfected

with control-siRNA (siCtrl) or *RelA*-siRNA (siRelA) oligonucleotides for 24 hours and followed by treatment with 100 nM PEM or the vehicle control (PBS) for additional 72 hours. Cells were lysed and analyzed by qRT-PCR (top) or immunoblotting (bottom). Data are shown as means and s.d. for three independent experiments (n = 3).

Suppl. Figure S5

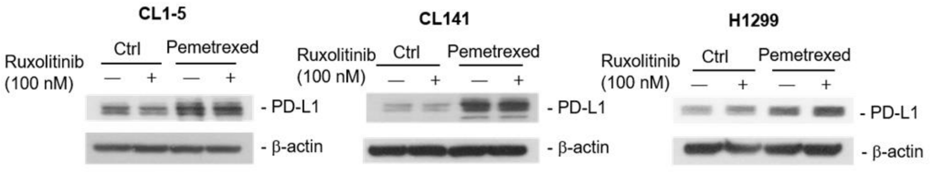

Figure S5.

The pemetrexed-induced PD-L1 expression is not mediated through IFN-γ-mediated JAK1/2 signaling pathway. CL1-5, CL141 and H1299 cells were pre-treated with 100 nM pemetrexed for 48 hours and incubated with 100 nM ruxolitinib (a JAK inhibitor) for additional 24 hours. Cells were then harvested and the levels of PD-L1 were determined by immunoblotting. The expression of β-actin serves as a loading control.

Lu C-S, *et al. J Immunother Cancer* 2020; 8:e001392. doi: 10.1136/jitc-2020-001392

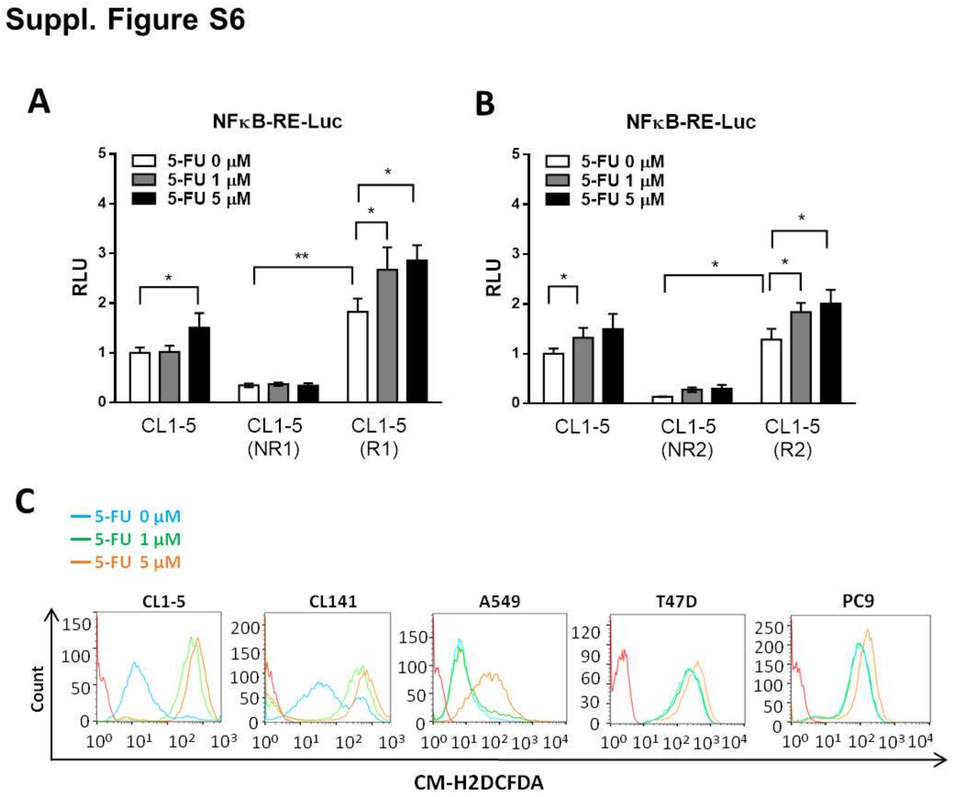

Figure S6.

(A, B) Transcriptional activation of a NF-κB reporter gene in 5-fluorouracil (5-FU)-treated parental and subclonal CL1-5 cells. NF-κB–Luc, a NF-κB responsive promoter–reporter construct, was transfected into each CL1-5 cell clone as indicated. The luminescence signal from the NF-κB–Luc reporter (firefly luciferase) was normalized to that of co-transfected pRL-TK vector (Renilla luciferase) to control for transfection efficiency and was expressed as relative light units (RLU). Data are shown as means and s.d. for three independent experiments (n = 3). (C) CL1-5, CL141, A549, T47D, or PC9 cells were treated with 5-FU (0, 1, or 5 μM) for 24 hours and followed by incubation with the DCFDA fluorogenic dye (10 μM) for 30 min. The levels of ROS were assessed by flow cytometry.

Lu C-S, *et al. J Immunother Cancer* 2020; 8:e001392. doi: 10.1136/jitc-2020-001392

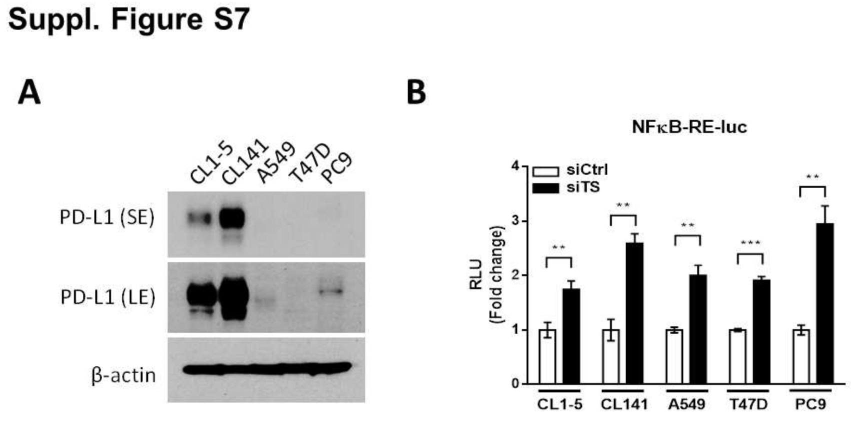

Figure S7.

(A) Western blot analysis for endogenous levels of PD-L1 in NSCLC cell lines (CL1-5, CL141, A549, and PC9) and breast cancer cell line (T47D). (LE, long exposure; SE, short exposure). (B) Transcriptional activation of a NF-κB reporter gene in NSCLC cell lines (CL1-5, CL141, A549, and PC9) and breast cancer cell line (T47D). NF-κB–Luc, a NF-κB responsive promoter–reporter construct, was transfected into each cell lines as indicated. The luminescence signal from the NF-κB–Luc reporter (firefly luciferase) was normalized to that of co-transfected pRL-TK vector (Renilla luciferase) to control for transfection efficiency and was expressed as relative light units (RLU). Data are shown as means and s.d. for three independent experiments (n = 3).

Suppl. Figure S8

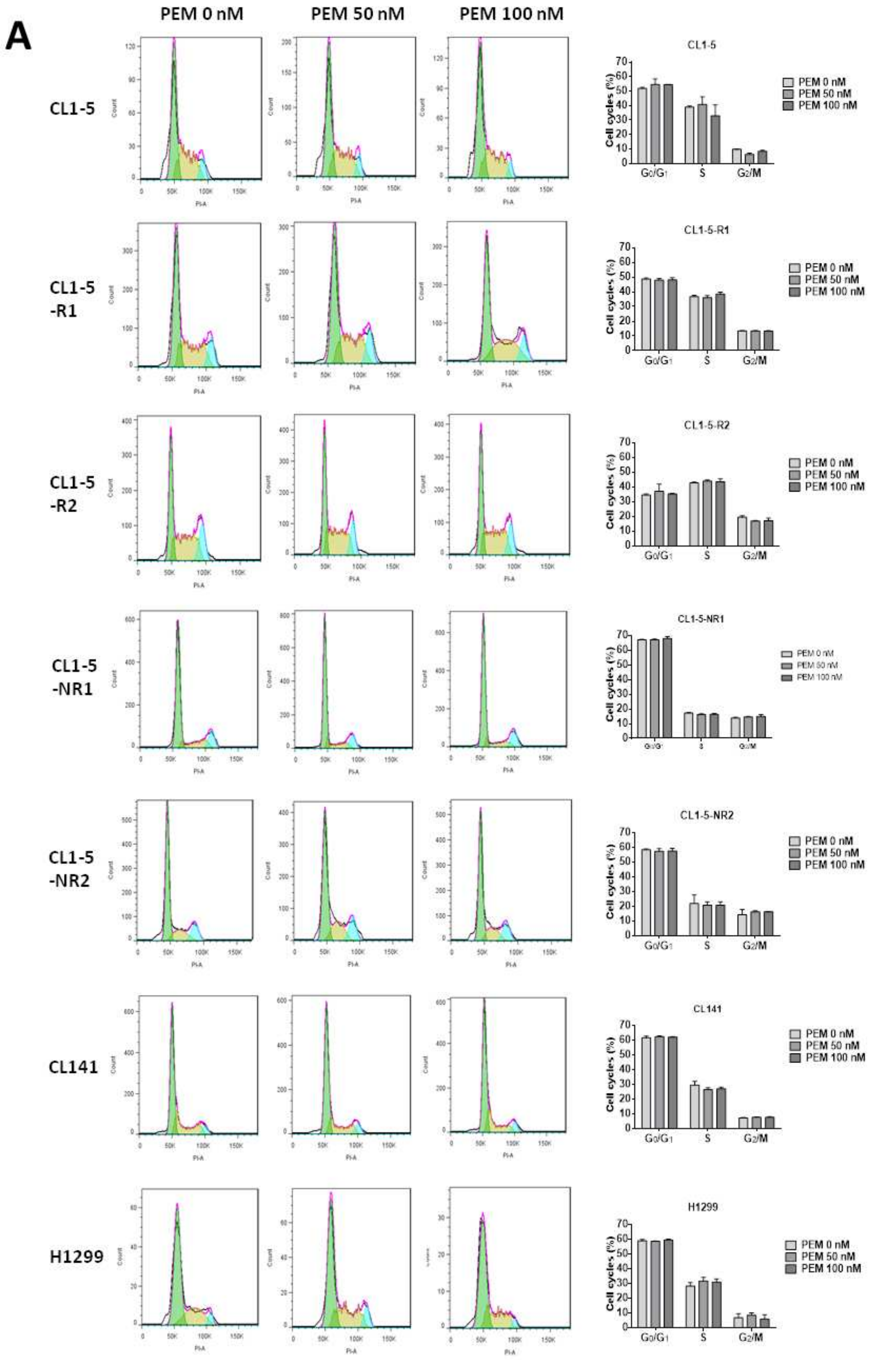

Lu C-S, et al. J Immunother Cancer 2020; 8:e001392. doi: 10.1136/jitc-2020-001392

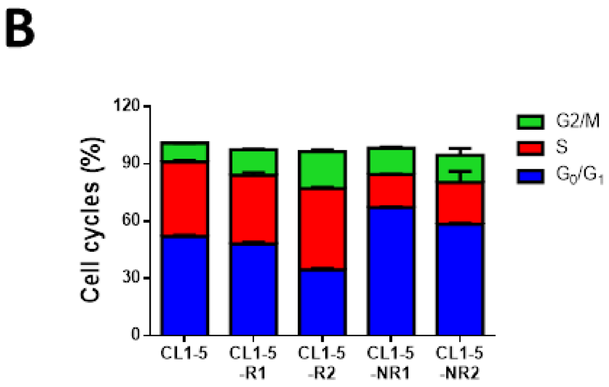

Figure S8.

(A) Analysis of the cell-cycle progression of pemetrexed (PEM)-treated CL141, H1299, CL1-5, and subclonal CL1-5 cells. Cells were seeded into six-well plates at a density of 3×10^5 cells per well in 6-cm dish. After one day, the cells were treated with PEM (0, 50, 100 nM) for 48 hours. At the end of the experiments, adherent cells were trypsinized and washed with PBS, and fixed by dropwise addition of 70% ice-cold ethanol at 4 °C overnight. The fixed cells were washed with PBS and incubated with 100 mg/ml RNase A and propidium iodide (40 mg/ml) at room temperature for 30 min. Cells were collected and analyzed with flow cytometry. Cell cycle distribution was analyzed using Flowjo software. (B) The cell cycle distribution of parental CL1-5, CL1-5-R1, CL1-5-R2, CL1-5-NR1 and CL1-5-NR2 cells. Data are shown as means and s.d. for three independent experiments (n = 3).

Lu C-S, *et al. J Immunother Cancer* 2020; 8:e001392. doi: 10.1136/jitc-2020-001392

Supplementary Materials and Methods

Cell viability assay

Cell viability was analyzed by Sulforhodamine B assay according to the manufacturer's instructions. Sulforhodamine B sodium salt (SRB) was purchased from Sigma-Aldrich (catalog number: S1402) and dissolved in 1% (vol/vol) acetic acid. Briefly, 5×10^3 cells per well were cultured in 96-well culture plates for overnight. The culture medium was replaced with fresh medium containing variant drugs at concentrations of 0.001–10 μM and cultured for 72 hours. Without removing the culture medium, the ice-cold Trichloroacetic acid was added to each well in a final concentration of 10% (wt/vol) and incubated for 30 min. Plates were then washed twice and left to air dry at room temperature (RT). Cells were later stained by 0.4% (wt/vol) SRB solution and incubated at RT for 30 min. The excess dye was then removed by washing repeatedly with 1% acetic acid. Plates were then left to air dry at RT. Protein-bound dye was dissolved in 10 mM Tris-base solution and the absorbance of each sample at O.D. 510 nm was measured using a microplate reader. Cell growth curves were plotted using GraphPad software.

Immunoblot analysis and antibodies

Whole cell lysates were collected and subjected to immunoblotting as previously described[1]. The primary antibodies used for immunoblotting were anti-PD-L1 (E1L3N, Cell Signaling Technology), anti-thymidylate synthase (D5B3, Cell Signaling Technology), anti-NF-κB p65 (phospho S536) (ab86299, Abcam),

Lu C-S, *et al. J Immunother Cancer* 2020; 8:e001392. doi: 10.1136/jitc-2020-001392

anti-NF-κB p65 (sc-109, Santa Cruz Biotechnology), rabbit anti-IKB-alpha (phospho S32) antibody (ab92700, Abcam), anti-IκB-α (FL) (sc-847, Santa Cruz Biotechnology), and anti-β-actin antibody (AC-15, Sigma-Aldrich). Primary antibody to human CD274 (B7-H1, PD-L1) (#329709) used in the coculture system and T cell-mediated killing assays was purchased from BioLegend.

Flow cytometry analysis

Cells treated with chemotherapeutic agents for 48 hours were trypsinized and washed with ice-cold PBS, then incubated with PE-conjugated antibodies against human or mouse PD-L1 (Anti-Human CD274(B7-H1)-PE, Clone: M1H1, Thermo Fisher Scientific), or relative isotype control antibodies (Mouse IgG1 kappa Isotype Control-PE, eBioscience) for 1 hour at 4°C. Cells were washed three times with ice-cold PBS and subjected to Fluorescence-activated cell sorting (FACS) analysis. The mean fluorescent intensity (MFI) obtained by flow cytometry indicates the expression level of PD-L1 on the cell surface. Relative MFI was normalized to that of in the vehicle control (PBS).

Real-time reverse transcriptase polymerase chain reaction (qRT-PCR)

Total RNA was extracted using TRIzol reagent (Invitrogen) and cDNA was prepared using the VersorogDNA synthesis kit (Thermo Fisher Scientific). qRT-PCR was performed using an ABI PRISM 7500 system (Applied Biosystems). Each reaction contained 50ng cDNA with a TaqMan 2× Universal Master Mix (Applied Biosystems). Primer sets for human CD274 (Hs00204257_m1), human GAPDH

(Hs99999905_m1), mouse CD274 (Mm03048248_m1), mouse IL-2 (Mm00434256_m1), mouse IFNγ (Mm01168134_m1) and TNFα (Mm00443258_m1), and mouse GAPDH (Mm99999915_g1) were purchased from Applied Biosystems. Normalization was performed using GAPDH as an internal control, and relative gene expression was calculated using the comparative 2(-ΔΔrm) method[2].

RNA interference

Transient knockdown experiments were conducted using human siRNA-SMARTpool for TS and RelA (Thermo Scientific). CL1-5 or CL141 cells were transfected with the indicated siRNAs using Lipofectamine RNAiMAX transfection reagent (Invitrogen) according to the manufacturer's protocols. The total amount of siRNA-SMARTpool for transfection was kept constant by the addition of the control siRNA (siCtrl). Cell lysates were harvested after 72 hours post-transfection.

Luciferase reporter assay

Cells were seeded in 12-well plates at a concentration of 5×10^4 per well. On the next day, cells were co-transfected with 100ng of pGL4.32/luc2P/NF-κB-RE/Hygro vector (Promega) and 2ng of the Renilla luciferase plasmid (phRL-TK, Promega) by lipofectamine 2000 (Invitrogen) according to manufacturer's instructions. After transfection, cells were treated with various concentrations of pemetrexed for 72 hours. Cells were harvested and the firefly and Renilla luciferase activities were determined using a dual-light luciferase reporter assay system (Promega).

Immunohistochemical staining

Two-μm-thick consecutive sections were cut from paraffin-embedded tissue blocks and floated onto glass slides. The slides were incubated at 65°C for 1 hour, deparaffinized in xylene, rehydrated in graded ethanol solutions, and boiled in Trilogy reagent (Cell Marque) for 10 min for antigen retrieval. After washing with PBS, the slides were immersed in 3% hydrogen peroxide for 10 min to suppress endogenous peroxidase activity. The sections were washed three times with PBS and incubated with anti-PD-L1 antibody (*Spring Bioscience*) at RT for 1 hour. Samples were washed three times again with PBS and incubated with biotinylated secondary antibody (Dako) for 25 min. After the washing step, slides were treated with horseradish peroxidase–conjugated streptavidin for 25 min. Peroxidase activity was developed with DAB (Dako), followed by counterstaining with hematoxylin. For assessing the expression of CD4 and CD8 in tumor-infiltrating lymphocytes, the sections were incubated overnight at 4℃ with 1:100 dilutions of primary antibodies against CD4 (Thermo Scientific) and CD8 (Thermo Fisher). Signals were amplified using an ImmPRESS HRP anti-rat IgG, mouse adsorbed (peroxidase) polymer detection kit (Vectors), with peroxidase activity developed with DAB peroxidase substrate (Vectors).

References:

1. Lin CW, Wang LK, Wang SP, et al. Daxx inhibits hypoxia-induced lung cancer cell metastasis by suppressing the HIF-1α/HDAC1/Slug axis. *Nat Commun* 2016;7:13867.

2. Schmittgen TD, Livak KJ. Analyzing real-time PCR data by the comparative C(T) method. *Nat Protoc* 2008;3:1101-1108.

Lu C-S, *et al. J Immunother Cancer* 2020; 8:e001392. doi: 10.1136/jitc-2020-001392
